# Upregulation of 5′-terminal oligopyrimidine mRNA translation upon loss of the ARF tumor suppressor

**DOI:** 10.1038/s41598-020-79379-8

**Published:** 2020-12-17

**Authors:** Kyle A. Cottrell, Ryan C. Chiou, Jason D. Weber

**Affiliations:** 1 Division of Molecular Oncology, Department of Medicine, Siteman Cancer Center, Washington University School of Medicine, 660 South Euclid Avenue, Campus, Box 8069, Saint Louis, MO 63110 USA; 2 Department of Cell Biology and Physiology, Siteman Cancer Center, Washington University School of Medicine, Saint Louis, MO USA

**Keywords:** Tumour-suppressor proteins, Translation

## Abstract

Tumor cells require nominal increases in protein synthesis in order to maintain high proliferation rates. As such, tumor cells must acquire enhanced ribosome production. How the numerous mutations in tumor cells ultimately achieve this aberrant production is largely unknown. The gene encoding ARF is the most commonly deleted gene in human cancer. ARF plays a significant role in regulating ribosomal RNA synthesis and processing, ribosome export into the cytoplasm, and global protein synthesis. Utilizing ribosome profiling, we show that ARF is a major suppressor of 5′-terminal oligopyrimidine mRNA translation. Genes with increased translational efficiency following loss of ARF include many ribosomal proteins and translation factors. Knockout of *p53* largely phenocopies ARF loss, with increased protein synthesis and expression of 5′-TOP encoded proteins. The 5′-TOP regulators eIF4G1 and LARP1 are upregulated in *Arf-* and *p53-*null cells.

## Introduction

Accelerated cellular division and macromolecular growth of tumors is dependent on robust protein synthesis. To meet this need, ribosome biogenesis and translation rates are frequently elevated in cancer cells^1^. Increased translation in cancer cells is often driven by activation of the mTORC1 pathway^1^.

Loss of the gene encoding the ARF tumor suppressor is the most common copy number variation in cancer^2^. ARF is expressed in response to oncogenic stimuli—including the overexpression of Myc, oncogenic Ras and chronic activation of the mTORC1-pathway^3,4^. The canonical function of ARF is to stabilize p53 by sequestering MDM2 in the nucleolus^4–6^. In addition to its canonical role, ARF suppresses global protein synthesis and ribosome biogenesis by regulating rRNA transcription and processing^7–16^. ARF also regulates the translation of specific mRNAs, including VEGFA and DROSHA^10,17^.

While ARF is known to regulate ribosome biogenesis, the p53-MDM2 axis senses dysregulation of ribosome biogenesis^18^. Free ribosomal proteins, such as RPL11, bind to MDM2 and lead to stabilization of p53^18^. In addition, p53 is known to repress the activity of mTORC1 during genotoxic stress^19^.

The synthesis of ribosomal proteins is tightly regulated by the mTORC1 pathway^20^. Multiple components of the translation machinery, including many ribosomal proteins and some translation factors contain a 5′-terminal oligopyrimidine (5′-TOP) motif at the beginning of their mRNAs^21,22^. mTORC1 has been shown to enhance 5′-TOP mRNA translation via two mechanisms; phosphorylation of 4EBP1 and/or LARP1^23–28^. Phosphorylation of 4EBP1 by mTORC1 prevents 4EBP1 from disrupting the interaction of eIF4E and the mRNA cap, and has been shown to selectively enhance 5′-TOP mRNA translation^25,26^. However, the role of 4EBP1 in regulating 5′-TOP mRNA translation has been disputed, as it has been shown that 4EBP1 is dispensable for regulation of 5′-TOP mRNAs under some conditions^29^. LARP1 has a dual-role in regulating the translation of 5′-TOP mRNAs—acting as a repressor and enhancer^24^. As a repressor of 5′-TOP mRNA translation, LARP1 disrupts eIF4E binding to the 5′ end of the mRNA, preventing formation of the eIF4F complex^24^. Activation of the mTORC1 pathway leads to phosphorylation of LARP1 by S6-kinase and mTORC1^24^. When phosphorylated, LARP1 no longer binds to the 5′UTR to repress translation. Phosphorylated LARP1 instead binds the 3′UTR of 5′-TOP mRNAs and enhances their translation^24^.

Here, we show that ARF selectively regulates the synthesis of ribosomal proteins and translation factors containing 5′-TOP motifs within their mRNAs. Knockdown or knockout of ARF caused increased translation of many 5′-TOP mRNAs. This effect of ARF-loss was dependent on p53 expression. Knockout of *p53* caused a similar increase in the expression of some 5′-TOP mRNA encoded proteins. Finally, we observed upregulation of many regulators of 5′-TOP mRNA translation following loss of ARF or p53.

## Results

### Increased protein synthesis following loss of ARF

**Figure 1:**
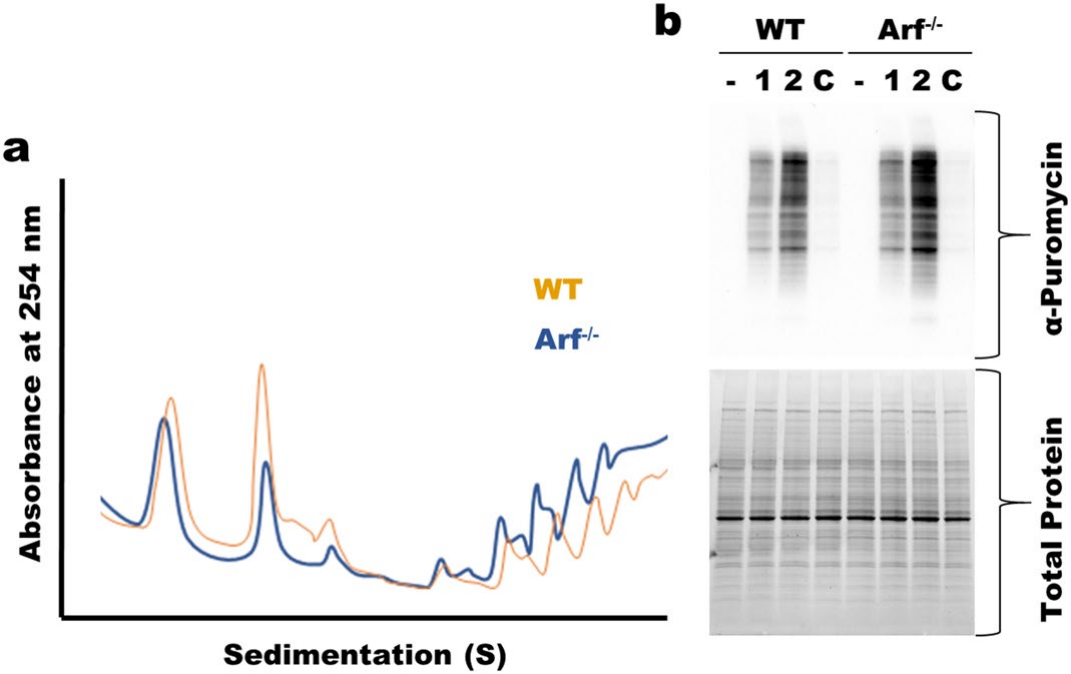
Upregulation of translation following loss of ARF. (**a**) Polysome profiling showing increased polysome peaks in *Arf*^*−/−*^ MEFs (**b**) Puromycin translation assay showing increased protein production in *Arf*^*−/−*^ MEFs. – untreated, 1 and 2 treatment with puromycin for 1 or 2 min, C indicates cells that were treated with cycloheximide prior to puromycin treatment for 2 min.

ARF has been previously identified as a regulator of global protein synthesis^10,16^. To confirm these previous findings, we assessed global protein synthesis using wildtype (WT) and *Arf*^*−/−*^ mouse embryonic fibroblasts (MEFs). The *Arf*^*−/−*^ mouse has been described previously^30^. In the *Arf*^*−/−*^ mice, exon1β of *Cdkn2a*, the gene encoding both *Arf* (p19) and *Ink4a* (p16), has been replaced with a neo cassette. This deletion has no effect on INK4A expression while providing a complete knockout of *Arf*^30^. Polysome profiling of WT and *Arf*^*−/−*^ MEFs revealed a shift of rRNA mass towards polysomes in *Arf*^*−/−*^, indicating more translation in those cells, Fig. 1a and Supplemental Fig. 1. This result is consistent with previous studies^10,16^. We further confirmed this observation by measuring the rate of puromycin incorporation into nascent peptides in WT and *Arf*^*−/−*^ MEFs. Puromycin is a translation elongation inhibitor that is incorporated into nascent peptide during protein synthesis^31^. Using a puromycin antibody it is possible to detect puromyclylated peptides. We observed an increase in puromycylated proteins in *Arf*^*−/−*^ MEFs compared to WT MEFs, Fig. 1b, further confirming elevated protein synthesis in those cells.

### Increased translation efficiency of 5′-TOP mRNAs upon ARF depletion

**Figure 2:**
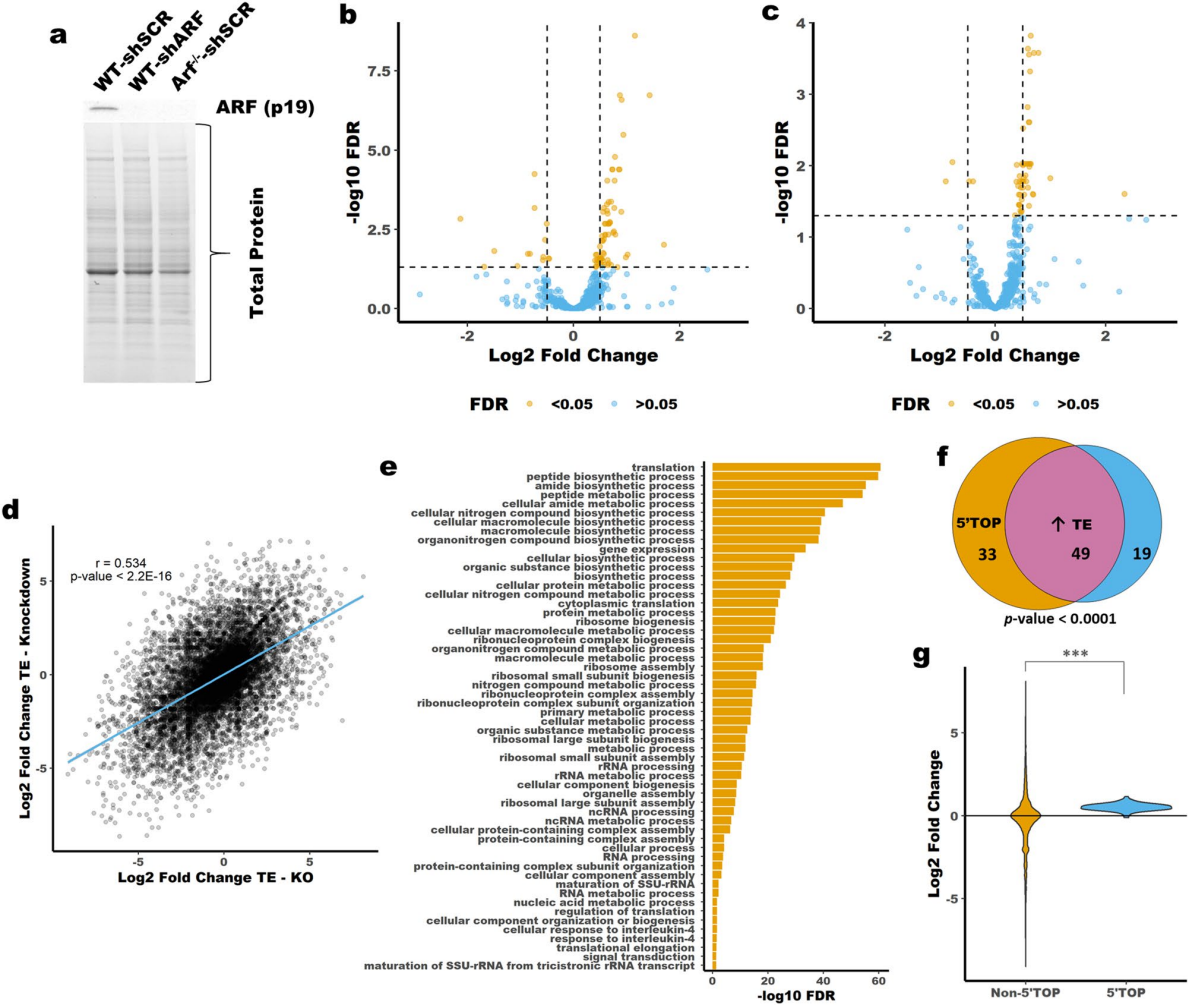
Ribosome profiling reveals upregulation of 5′-TOP mRNA translation following loss of ARF. (**a**) Immunoblot showing knockdown and knockout of *Arf* in MEFs. (**b**) Volcano plot showing Log2 Fold Change of TE between WT-shSCR and *Arf*^*−/−*^ -shSCR MEFs or WT-shSCR and WT -shARF MEFs (**c**) Vertical lines are Log2 Fold Change of ± 0.5. Horizontal line is FDR corrected *p*-value of 0.05. (**d**) Correlation between Fold Change of TE following ARF knockout (x-axis) or ARF knockdown (y-axis). (**e**) Geno ontology terms associated with genes that have increased TE following ARF knockout. (**f**) Venn-diagram showing overlap between mRNAs with increased TE and the presence of a 5′-TOP motif. (**g**) Violin plot showing the TE of mRNAs known to contain a 5′-TOP motif versus those that are not known to contain the motif^21^. *** *p*-value < 0.00001; Hypergeometric test (**f**) or two-tailed t-test (**g**).

Previously ARF has been identified to not only regulate translation globally but to specifically regulate the translation of a subset of mRNAs^10,17^. To map global translational regulation by ARF we employed ribosome profiling to measure changes in translation following ARF depletion. We performed ribosome profiling and RNAseq for WT MEFs transduced with either shSCR or shARF in addition to *Arf*^*−/−*^ MEFs transduced with shSCR, Fig. 2a. The shRNA used to knockdown ARF has been described previously and has no effect on INK4A expression^16^. Replicate-to-replicate comparisons show strong correlations between RNAseq or ribosome profiling replicates (Supplemental Fig. 2b). We used DESeq2 to identify differential translation efficiency (TE) as described previously^32^. Most mRNAs showed no significant difference in TE upon loss of ARF, Fig. 2b,c. However, a subset of mRNAs showed a slight but significant increase in translation following ARF depletion by either knockdown with shARF or knockout of *Arf*. There is a significant correlation between fold change of TE following ARF knockdown and knockout, Fig. 2d. Gene ontology (GO) analysis of the genes with increased TE following knockout of *Arf* identified multiple GO terms associated with translation, Fig. 2e. A closer look at the genes with the biggest increase in TE revealed many ribosomal proteins and translation factors. It has been well established that most ribosomal proteins and many translation factors contain a 5′-TOP motif^21,22^. Analysis of our ribosome profiling results revealed that many of the mRNAs with increased TE are known to contain a 5′-TOP motif, Fig. 2f. ^21^. Furthermore, mRNAs known to contain a 5′-TOP motif show a general increase in TE following knockout of *Arf*, Fig. 2g.

**Figure 3:**
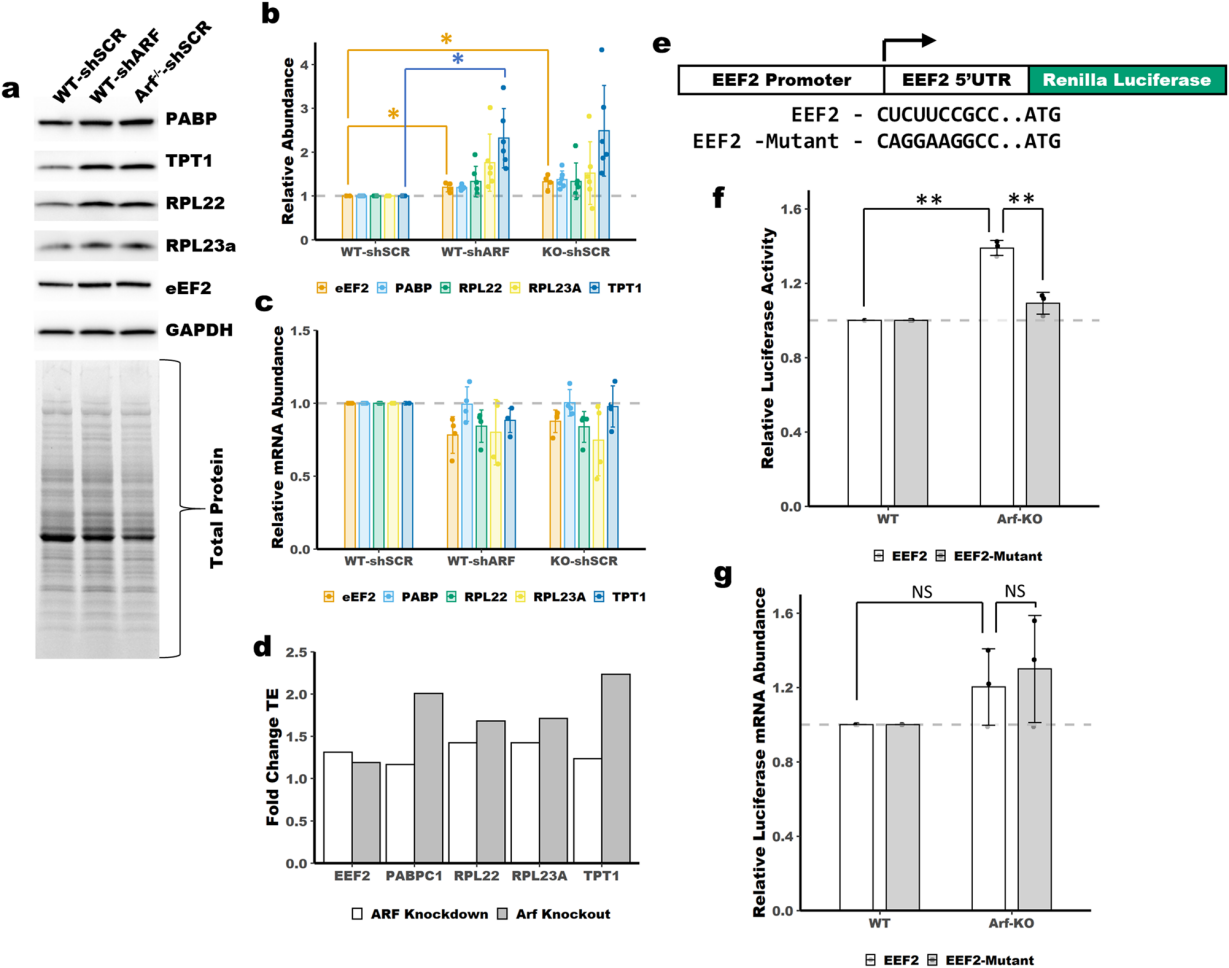
Upregulation of 5′-TOP mRNA translation following loss of ARF. (**a**) Immunoblot analysis showing increased protein abundance of PABP, TPT1, RPL23A, EEF2 and RPL22. (**b**) Quantitation of blots in panel (**a**). Total protein was used for normalization. Mean ± SD, n = 6. (**c**) qPCR shows no change in mRNA expression of 5′-TOP mRNAs. Mean ± SD, n = 4. (**d**) The average fold change of TE from two ribosome profiling replicates is shown for PABPC1, TPT1, RPL23A, EEF2 and RPL22. (**e**) Schematic of the luciferase reporters used in panels (**f**,**g**). (**f**) Luciferase activity of a 5′-TOP reporter is increased in *Arf*^*−/−*^ MEFs (Arf-KO). Luciferase activity was normalized to firefly luciferase transfection control and set relative to WT. Mean ± SD, n = 3. (**g**) qPCR shows no increase in mRNA expression of the 5′-TOP reporter. Normalized to firefly luciferase transfection control. Mean ± SD, n = 3. *, p-value < 0.05, ** *p*-value < 0.01; two-tailed t-test with Bonferoni correction.

To validate our ribosome profiling findings, we assessed the mRNA and protein abundance of several 5′-TOP genes in MEFs following depletion of ARF. By immunoblot we observed increased protein abundance for the 5′-TOP genes PABPC1, TPT1, EEF2, RPL23A and RPL22, Fig. 3a,b and Supplemental Figs. 3, 4. The increased protein abundance measured by immunoblot was generally consistent with the increased TE measured by ribosome profiling, Fig. 3d. The antibody used for PABPC1 was raised against a peptide from PABPC1, but may cross-react with other PABPCs, as such we have labeled this blot and others ‘PABP’. Because the expression of PABPC1 is much higher than that of the other PABPCs (Supplemental Dataset) it likely that most of the immunoblot signal is coming from PABPC1. The expression of PABPC1, TPT1, EEF2, RPL23A and RPL22 was unchanged at the mRNA level, Fig. 3c. Combined with our ribosome profiling results these data support increased translation of 5′-TOP mRNAs in ARF-null MEFs. To further validate these findings, we utilized a previously described 5′-TOP reporter^26^. The reporter contains the promoter and 5′-UTR of the 5′-TOP gene EEF2, Fig. 3e. The 5′-TOP reporter and a control that has a mutated 5′-TOP motif were transfected in parallel into WT or *Arf*^*−/−*^ MEFs along with a firefly luciferase control. We observed increased luciferase activity for our 5′-TOP reporter in *Arf*^*−/−*^ MEFs, Fig. 3f. This contrasts with the 5′-TOP-mutant reporter which had consistent activity between WT and *Arf*^*−/−*^ MEFs. Analysis of the mRNA abundance of the 5′-TOP reporter revealed no change in mRNA expression in *Arf*^*−/−*^ MEFs relative to WT, Fig. 3g. This indicates that the increased luciferase activity observed for the 5′-TOP reporter is caused by increased translation efficiency—not transcription or RNA stability. To provide further evidence we generated a second reporter system; the promoter and 5′UTR from *Rpl23a*, a 5′-TOP containing gene that showed increased TE upon ARF depletion in our ribosome profiling data, was cloned upstream of firefly luciferase, Supplemental Fig. 4b. The RPL23A reporter showed increased luciferase activity in ARF knockdown and knockout MEFs relative to WT, Supplemental Fig. 4c,d. Together these data confirm increased translation efficiency of 5′-TOP containing mRNAs following loss of ARF.

### Increased translation efficiency of 5′-TOP mRNAs upon ARF loss is dependent on p53

**Figure 4:**
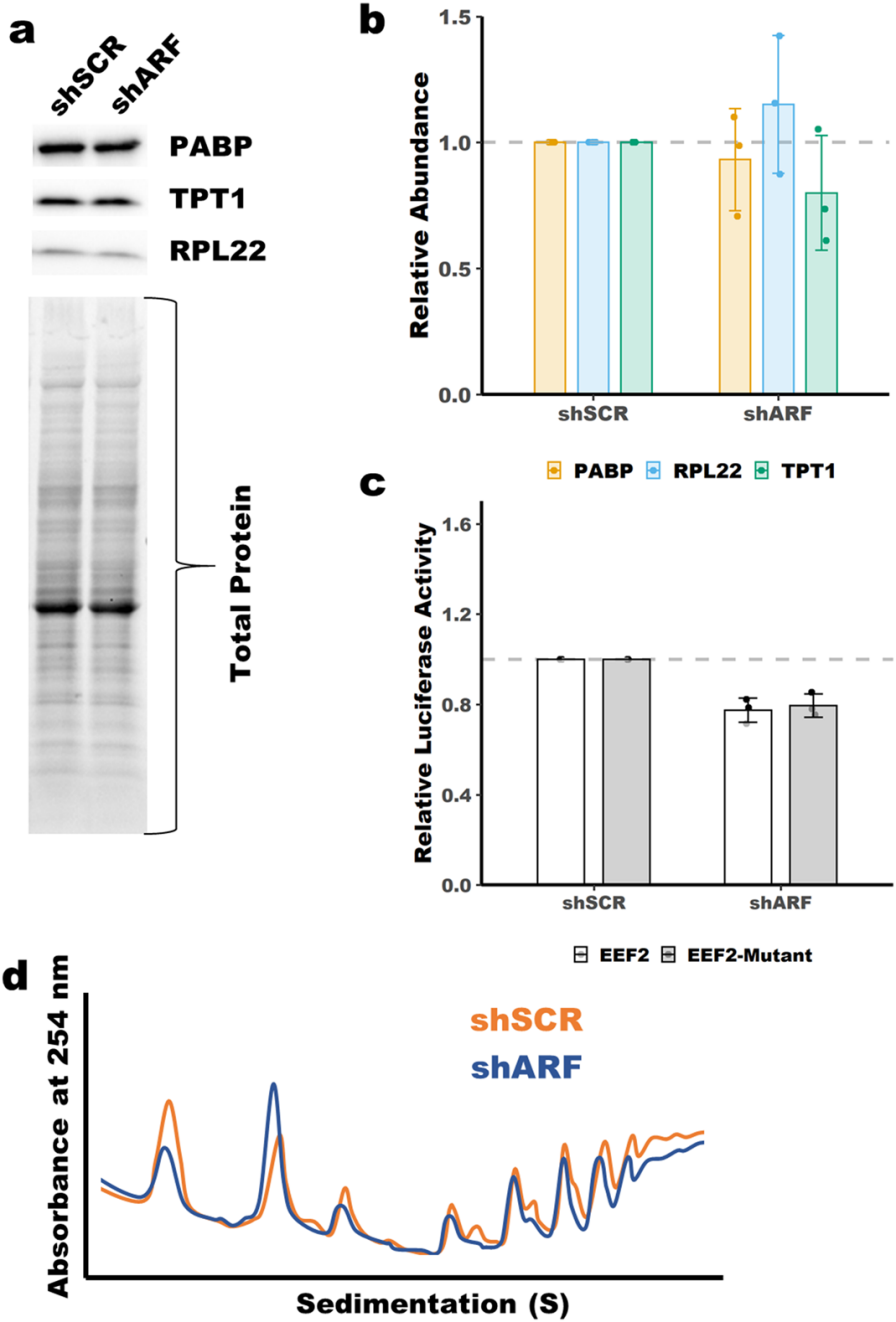
5′-TOP expression is unaffected by ARF knockdown in *p53*^*−/−*^ MEFs. (**a**) Immunoblot analysis showing no change in protein abundance of PABPC1, TPT1 and RPL22 following ARF knockdown in *p53*^*−/−*^ MEFs. (**b**) Quantitation of blots in panel (**a**). Total protein was used for normalization. Mean ± SD, n = 3. (**c**) No change in luciferase activity of a 5′-TOP reporter following ARF knockdown in *p53*^*−/−*^ MEFs. Luciferase activity was normalized to *Renilla* luciferase transfection control and set relative to shSCR. Mean ± SD, n = 3. (**d**) Polysome profiling showing no change in polysome peaks in *p53*^*−/−*^ MEFs following knockdown of ARF.

ARF is a well-known activator of p53^4,5^. To test whether p53 is required for ARF-dependent regulation of 5′-TOP mRNA translation we performed some of the experiments described above in *p53*^*−/−*^ MEFs. Knockdown of ARF in *p53*^−/−^ MEFs had no effect on protein abundance of the evaluated 5′-TOP genes, Fig. 4a,b and Supplemental Fig. 5b. Furthermore, knockdown of ARF had no effect on the translation of the EEF2 5′-TOP reporter or the RPL23A reporter, Fig. 4c and Supplemental Fig. 5c. There was no difference in polysome abundance between shSCR and shARF transduced *p53*^−/−^ MEFs indicating no changes in total protein synthesis, Fig. 4d and Supplemental Fig. 5a. However, there were some changes in the abundance 40S and 60S ribosomal subunits based on variations in peak heights. This observation mirrored that seen following knockout of *Arf* in WT MEFs. These findings indicate that p53 is required for ARF-dependent translational regulation of 5′-TOP mRNAs.

### Loss of ARF modestly affects the expression or activity of 5′-TOP regulators

**Figure 5:**
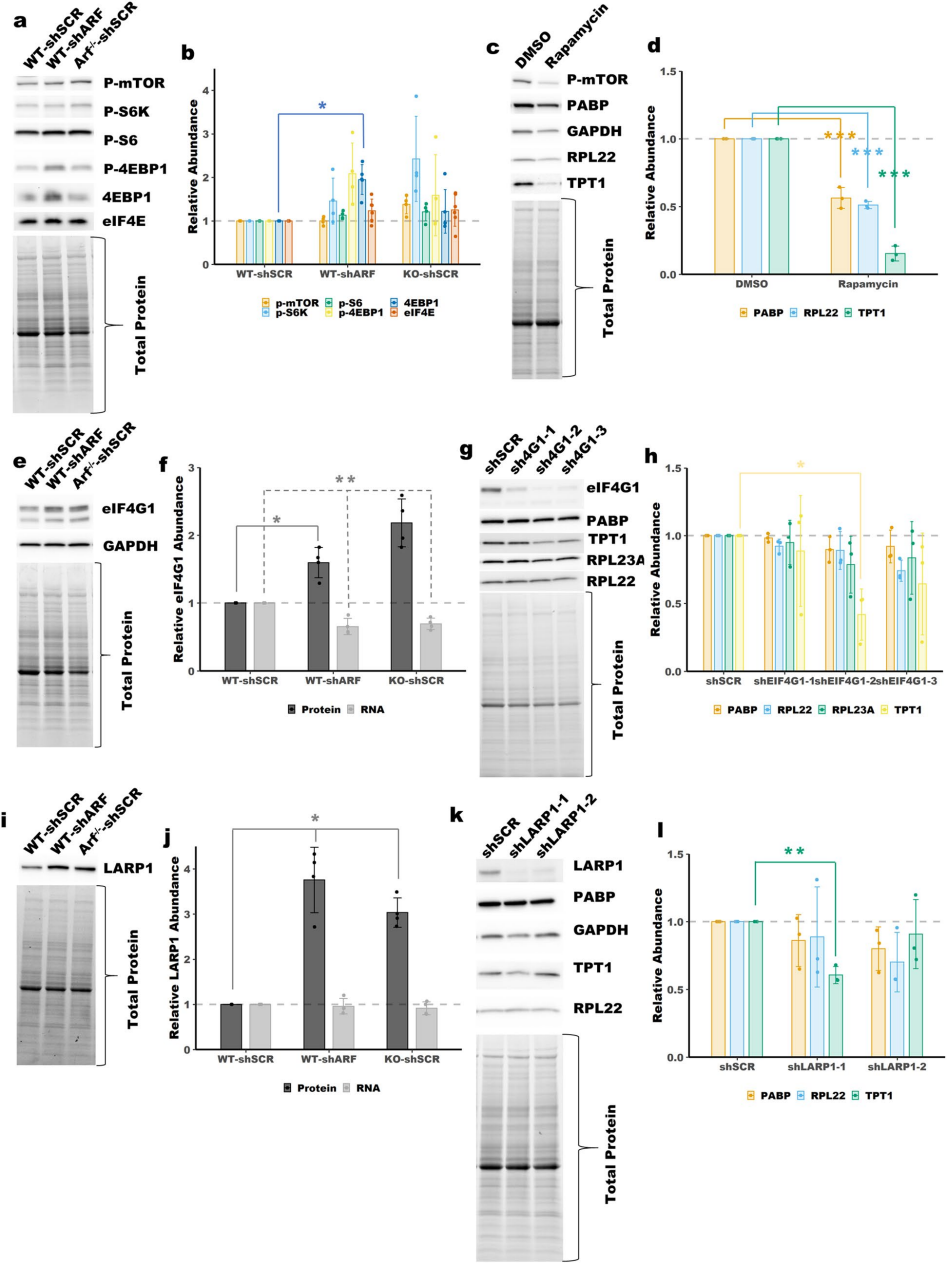
The expression and activity of 5′-TOP mRNA regulators following loss of tumor suppressors. (**a**) Immunoblot analysis showing activity of mTORC1 in WT and ARF-null MEFs and quantified in (**b**). p-mTOR = p-S2484, p-S6K = p-T389 (p70), p-S6 = p-S240/S244, p-4EBP1 = p-T37/T46. Total protein was used for normalization. Mean ± SD, n = 4–5 (**c**) Immunoblot analysis showing expression of 5′-TOP mRNA encoded proteins following treatment with rapamycin (Selleck Chemicals) and quantified in (**d**). *Arf*^*−/−*^ MEFs were treated with 5 nM rapamycin for 4 days. Total protein was used for normalization. Mean ± SD, n = 3. (**e**) Immunoblot analysis showing increased expression of eIF4G1 in *ARF*-null MEFs. **f** Quantitation of blot in (**e**) and qPCR for eIF4G1, total protein was used for normalization of immunoblot, Mean ± SD, n = 4. (**g**) Immunoblot analysis showing the effect of eIF4G1 knockdown on 5′-TOP gene expression. **h** Quantitation of blot in (**g**), total protein was used for normalization of immunoblot, Mean ± SD, n = 3. (**i**) Immunoblot analysis showing increased expression of LARP1 in *ARF*-null MEFs. (**j**) Quantitation of blot in **i** and qPCR for LARP1, total protein was used for normalization of immunoblot, Mean ± SD, n = 4. (**k**) Immunoblot of *Arf*^*−/−*^ MEFs following knockdown of LARP1 with two different shRNAs. (**l**) Quantitiation of blot in (**k**), total protein was used for normalization of immunoblot, Mean ± SD, n = 3. *, **, *** *p*-value < 0.05, < 0.01, < 0.001; two-tailed t-test with Bonferoni correction.

The mTOR pathway is known to regulate 5′-TOP mRNAs through the phosphorylation of LAPR1, which changes LARP1 from a repressor of 5′-TOP mRNA translation to an enhancer^23,24,27,28^. LARP1 is phosphorylated by mTORC1 and the mTORC1 substrate S6-kinase^24^. Immunoblot analysis of mTORC1 pathway activation in WT and ARF-null MEFs revealed a slight, though inconsistent and non-significant increase in mTORC1 activity, Fig. 5a,b and Supplemental Fig. 7a. S6-kinase showed the largest increase, though statistically insignificant, in phosphorylation following ARF depletion, indicating LARP1 may be phosphorylated in those cells. However, inhibition of S6-kinase had no effect on the expression of 5′-TOP encoded proteins, Supplementary Fig. 9. This result is consistent with previous studies that have shown S6-kinase to be dispensable for regulation of 5′-TOP mRNA translation^33^. Interestingly, we observed increased phosphorylation of S6-kinase, but no increased phosphorylation of other mTORC1 markers following ARF-knockdown in *p53*^*−/−*^ MEFs, Supplemental Fig. 6a,b. Treatment with the mTOR inhibitor rapamycin was employed to assess the importance of mTORC1 pathway activity in *Arf*^*−/−*^ MEFs. Consistent with previous reports, cells treated with rapamycin show reduced mTOR autophosphorylation and reduced expression of the 5′-TOP encoded proteins RPL22, PABPC1 and TPT1, Fig. 5c,d ^34,35^. These results show that mTORC1 activity is required for maximal expression of 5′-TOP mRNAs in ARF-null MEFs.

mTORC1 has been shown to enhance 5′-TOP translation through phosphorylation of 4EBP1^25^. There was a slight but insignificant increase in p-4EBP1 levels upon ARF knockdown, but no change upon knockout of ARF, Fig. 5a,b and Supplemental Fig. 7a. However, there was also an increase in total 4EBP1 levels upon ARF knockdown, Fig. 5a,b and Supplemental Fig. 7b. As unphosphorylated 4EBP1 is a translational repressor, the increased expression of 4EBP1 upon ARF knockdown is unlikely to contribute to increased 5′-TOP translation. The expression of eIF4E was unchanged upon knockout or knockdown of ARF, Fig. 5a,b and Supplemental Fig. 7c-d.

Maximal translation of 5′-TOP mRNAs is dependent on eIF4G1 expression^26^. Immunoblot analysis revealed increased eIF4G1 expression in ARF-null MEFs, Fig. 5e,f and Supplemental Fig. 8. A similar increase was observed in *p53*^*−/−*^ MEFs following ARF-knockdown, Supplementary Fig. 6c,d. We attempted to overexpress eIF4G1 in WT MEFs via lentiviral transduction, but due to the size of the coding sequence the transduction efficiency was extremely low (data not shown). As such, we took the reverse approach and knocked-down eIF4G1 in *Arf* ^*−/−*^ MEFs. Knockdown of eIF4G1 caused a slight reduction of protein expression for several 5′-TOP genes at 4 days post transduction, Fig. 5g,h, and Supplemental Fig. 8b. These data suggest that increased eIF4G1 expression is unlikely to be driving increased translation of all 5′TOP mRNAs, but may have a role in regulating specific mRNAs like TPT1.

LARP1 is a known regulator of 5′-TOP mRNA translation^24,27,28^. Immunoblot analysis of WT and ARF-null MEFs showed increase protein abundance for LARP1, Fig. 5i,j and Supplemental Fig. 8c. There was no increase in LARP1 mRNA expression in ARF-null MEFs relative to WT MEFs, Fig. 5j. Knockdown of ARF in *p53*^*−/−*^ MEFs had no effect on LARP1 expression, Supplementary Fig. 5e,f. Given the dual role of LARP1 as both an enhancer and repressor of 5′-TOP mRNA translation^24^ it is possible that elevated expression of LARP1 in this context could drive 5′-TOP mRNA translation. However, knockdown of LARP1 had little to no effect on the expression of several 5′-TOP genes in *Arf*^*−/−*^ MEFs, Fig. 5k,l. Only one of the two shRNAs targeting LARP1 had a significant effect on expression but for only one of the 5′-TOP genes assessed – TPT1. These findings indicate that elevated LARP1 expression is not likely to be the driving force behind increased 5′-TOP mRNA translation following loss of ARF, though it may have a role in the regulation of specific 5′-TOP mRNAs.

### Loss of p53 causes upregulation of 5′-TOP mRNAs

**Figure 6:**
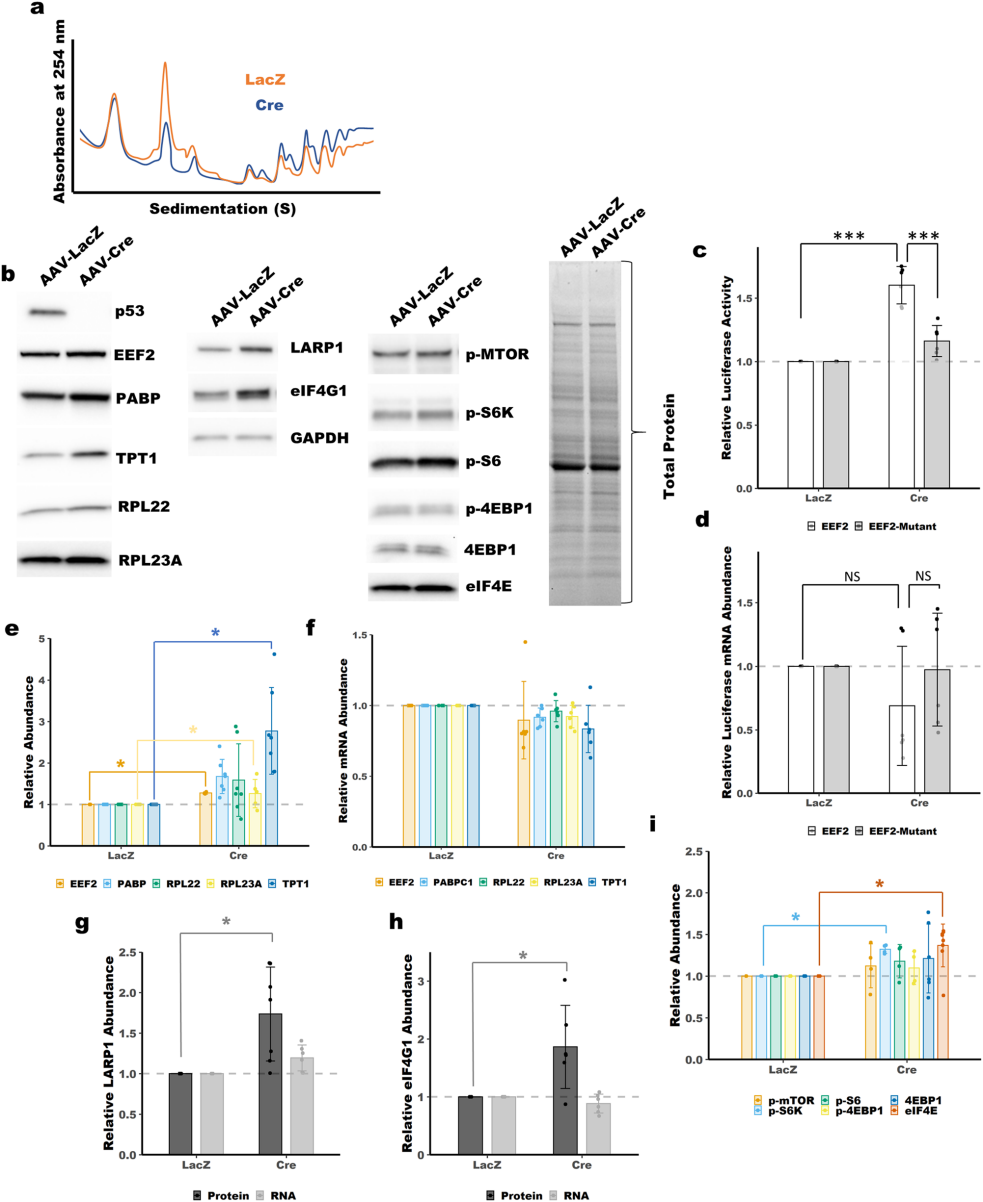
Upregulation of 5′-TOP mRNAs in *p53*^*−/−*^ MEFs. *p53*^fl/fl^ MEFs were transduced with control AAV-LacZ or AAV-Cre to knockout *p53*. (**a**) Polysome profiling showing increased polysome peaks following knockout of *p53* (Cre). (**b**) Immunoblot analysis showing protein abundance of 5′-TOP encoded proteins (TPT1, RPL22, RPL23A, EEF2 and PABPC1) and 5′-TOP regulators (eIF4G1, LARP1, eIF4E and mTORC1) following knockout of *p53* (AAV-Cre). (**c**) Luciferase activity of a 5′-TOP reporter is increased in *p53*^*−/−*^ MEFs. Luciferase activity was normalized to firefly luciferase transfection control and set relative to LacZ control. Mean ± SD, n = 3. (**d**) qPCR shows no increase in mRNA expression of the 5′-TOP reporter. Normalized to firefly luciferase transfection control. Mean ± SD, n = 3. (**e**) Quantitation of blots in panel (**b**) for TPT1, RPL22, RPL23A, EEF2 and PABPC1. Total protein was used for normalization. Mean ± SD, n = 3–7. (**f**) qPCR shows no change in mRNA expression of 5′-TOP mRNAs following knockout of *p53*. Mean ± SD, n = 6. (**g**) Quantitation of blot in (**b**) and qPCR for LARP1. Mean ± SD, n = 7(protein), 6(RNA). (**h**) Quantitation of blot in (**b**) and qPCR for eIF4G1. Mean ± SD, n = 6(protein), 6(RNA). (**i**) Quantitation of blot in (**b**) and for mTORC1 pathway. Mean ± SD, n = 4. *, **, *** *p*-value < 0.05, < 0.01, < 0.001; two-tailed t-test with Bonferoni correction.

Having observed a role for ARF in regulation 5′-TOP mRNA translation, we next sought to determine if p53, which is tightly coupled to ARF expression, has a similar role. Polysome profiling revealed increased translation in *p53*^*−/−*^ MEFs, as indicated by higher polysome peaks, Fig. 6a and Supplemental Fig. 10a-b. We used immunoblot analysis and qPCR to determine the protein and mRNA expression of some of the 5′-TOP genes studied above. We observed an increase in protein expression for three of the 5′-TOP genes (PABPC1, EEF2 and TPT1) in *p53*^*−/−*^ MEFs, there was no increase in RNA expression, Fig. 6b,e,f, and Supplemental Figs. 10 and 11. This is consistent with our findings from *ARF*-null MEFs. Analysis of the luciferase reporters described above showed increased activity for the EEF2 5′-TOP reporter and the RPL23A reporter in *p53*^*−/−*^ MEFs, Fig. 6c and Supplemental Fig. 10c, without any increase in mRNA expression, Fig. 6d and Supplemental Fig. 10d.

Consistent with *Arf* knockout, immunoblot analysis of WT and *p53*^*−/−*^ MEFs revealed an increase in LARP1 and eIF4G1 expression, Fig. 6b,g,h, and Supplemental Figs. 10–11. Immunoblot analysis of mTORC1 activity showed no changes in phosphorylation level of mTOR itself and only modest increases in phosphorylation of downstream pathway components following knockout of p53, Fig. 6b,I, and Supplemental Fig. 11. Knockout of *p53* had no effect on 4EBP1 expression or phosphorylation, Fig. 6b,i. Unlike knockout of *Arf*, we did observe increased expression of eIF4E, Fig. 6b,i, and Supplemental Fig. 11.

## Discussion

Together these data fit well with what is known about the functions of ARF in ribosome biogenesis. ARF is known to repress ribosome biogenesis by affecting rRNA transcription, processing and nuclear export^7–16^. Changes in 5′-TOP mRNA translation upon loss of *Arf* would help to balance concomitant changes in rRNA production by providing increases in the necessary ribosome subunit proteins. Increased translation of 5′-TOP mRNAs following loss of the ARF tumor suppressor provides a missing mechanism for the increased rates of translation and ribosome biogenesis seen in many human cancers^36^.

Previously, it has been shown that loss of ARF caused enhanced translation of two mRNAs: DROSHA and VEGFA^10,17^. We did not observe any significantly increased translation efficiency for either DROSHA or VEGFA following knockdown or knockout of ARF. One possible explanation for the discrepancy regarding VEGFA could be the cell line used. The VEGFA experiments were performed in established NIH3T3 and *Arf/p53* DKO MEFs with overexpression of ARF, not knockdown/knockout^17^.

While many of the functions of ARF in regulating ribosome biogenesis are p53-independent, we show here that the regulation of 5′-TOP mRNAs by ARF requires p53 expression. Furthermore, we show that loss of p53 partially phenocopies the effects of ARF depletion on 5′-TOP mRNA translation. These data suggest that canonical ARF-MDM2-p53 axis plays an important role in 5′-TOP mRNA translation. Interestingly, free ribosomal proteins, such as RPL11, are known to activate p53 through binding MDM2^18^. Elevated expression of 5′-TOP containing ribosomal proteins following loss of *p53* would complete a feedback loop. Recently it has been shown that the cyclin-dependent kinase CDK1 can phosphorylate LARP1 and 4EBP1, and has a general role in enhancing translation^37^. As p53 represses CDK1 activity through transcriptional upregulation of p21 (*Cdkn1a*), the ability of CDK1 to phosphorylate LARP1 and 4EBP1 could contribute to increased 5′-TOP translation following loss of p53 or ARF—though we did not see evidence of increased 4EBP1 phosphorylation (relative to total 4EBP1) following loss of p53 or ARF. In support of this hypothesis, we observed decreased RNA expression of p21 upon knockout of *Arf* (Supplementary Dataset). The connection between ARF, p53, p21, CDK1 and regulation of 5′-TOP translation deserves further investigation.

It is unclear what factors drive increased 5′-TOP mRNA translation following loss of ARF. We observed increased expression and activity for several regulators of 5′-TOP mRNA translation. Depletion of ARF had little effect on mTORC1 activity, though there was a slight and non-significant increase in phosphorylation of some pathway components. By inhibiting mTOR with rapamycin we showed that maximal expression of 5′-TOP encoded proteins requires mTOR activity. mTORC1 enhances 5′-TOP mRNA translation through phosphorylation of LARP1. LARP1 acts to balance 5′-TOP mRNA translation through inhibition of translation when dephosphorylated and enhancing translation when phosphorylated^24^. Dephosphorylated LARP1 binds the 5′-TOP motif and blocks binding of eIF4E, thus blocking assembly of the eIF4F complex^27^. A component of the eIF4F complex, eIF4G1 has also been shown to be required for 5′-TOP translation^26^. We observed increased expression of both eIF4G1 and LARP1 following depletion of ARF. Because of technical limitations described above, it is difficult to assess the direct role of eIF4G1 in regulating endogenous 5′-TOP mRNAs via overexpression in this system. Knockdown of eIF4G1 caused a slight and somewhat inconsistent reduction in 5′-TOP gene expression at the protein level. Knockdown of LARP1 in *Arf*^*−/−*^ MEFs had only modest effects on 5′-TOP mRNA expression, with one shRNA causing a significant reduction in the expression of the 5′-TOP mRNA encoded protein TPT1. This suggests that at least for TPT1, LARP1 may modestly enhance translation. Because phosphorylated LARP1 is known to enhance 5′-TOP mRNA translation, this result is consistent with active mTORC1 in *Arf*^*−/−*^ MEFs. Increased 5′-TOP mRNA translation could be driven by the combinatorial effect of small increases in eIF4G1 and LARP1 expression, as well as increased mTORC1 activity. A more intriguing possibility is that an unknown factor—downstream of ARF—controls 5′-TOP mRNA translation. Further work is needed to elucidate this pathway. Because many 5′-TOP encoded proteins are essential components of the translation machinery, it is important to understand how loss of ARF can drive 5′-TOP mRNA translation and global protein synthesis. A complete understanding of this pathway may lead to future therapeutic interventions aimed at reducing the protein synthesis capacity of ARF-null tumors.

Regulation of 5′-TOP mRNA translation independent of mTORC1 has been recently observed in mouse liver. Knockdown of the translation elongation factor eEF2 increased the translation efficiency of 5′-TOP containing mRNAs, without any change in mTORC1 activity^38^. The findings described here and those from knockdown of eEF2 in mouse liver suggest that there is another pathway regulating 5′-TOP mRNA translation.

One potential factor driving increased 5′-TOP translation in *p53*^*−/−*^ MEFs could be the cap-binding protein eIF4E. We observed a slight increase in eIF4E expression upon p53 knockout, however the same was not true following ARF depletion. The affinity of eIF4E for the mRNA cap can be influenced by cap-proximal sequences. In fact, 5′-TOP motif containing sequences are sensitive to reductions in eIF4E levels and eIF4E has a lower affinity for RNAs containing 5′-TOP motifs^39^. Future studies are needed to explore the connection between p53, eIF4E and 5′-TOP mRNA translation.

The observation that eIF4G1 and LARP1 are upregulated upon ARF depletion deserves further investigation. Both eIF4G1 and LARP1 were upregulated at the protein level upon loss of ARF or p53, while there was no change at the mRNA level. From our ribosome profiling data, we know that neither eIF4G1 nor LARP1 are translationally upregulated. These data suggest that the half-life of LARP1 and eIF4G1 proteins may be extended upon ARF or p53 depletion. Upregulation of LARP1 has been seen in many cancers^40–42^. Additionally, eIF4G1 is upregulated in many breast cancers^43,44^. As deletion of *Arf* is a common occurrence in cancer, exploring the co-occurrence of *Arf*-loss and increased LARP1 or eIF4G1 expression seems warranted.

## Materials and methods

### Cell culture

Mouse embryonic fibroblasts (MEFs) were harvested previously from WT (B6129SF2/J), *Arf* ^*−/−*^ (B6.129X1-*Cdkn2a*^*tm1Cjs*^/KaiJ), *p53*^*flox/flox*^ (FVB.129-*Trp53*^*tm1Brn*^). MEFs were cultured in Dulbecco’s modified Eagle’s medium (DMEM) (Hyclone) with 10% fetal bovine serum (Invitrogen), 2 mM glutamine (Hyclone), 0.1 mM nonessential amino acids (Hyclone), 1 mM sodium pyruvate (Hyclone), and 2 μg/ml gentamicin (Invitrogen). Low passage MEFs, between 1–4 (WT or *p53f.*^*/f*^) and 1–7 (ARF^*−/−*^), were used for all experiments.

### Viral production and transduction

Lentivirus was produced by Lipofectamine 2000 (Inivitrogen) transfection of 293 T cells with pCMV-VSV-G, pCMV-ΔR8.2, and pLKO.1-puro for shRNAs. Virus was harvested 48 h post-transfection. Cells were transduced with lentivirus for 16 h in the presence of 10 µg/mL protamine sulfate. The cells were selected with puromycin at 2 µg/mL for two days. The sequences for the shRNA-scramble (shSCR) and shRNA-ARF (shARF) were described and validated previously^16^. LARP1 and eIF4G1 shRNAs were purchased from Millipore-Sigma: LARP1 shRNA-1: 5′-CCGGGAGTCTCCAAACTACCGAAATCTCGAGATTTCGGTAGTTTGGAGACTCTTTTTG-3′, LARP1 shRNA-2: 5′-CCGGGGACAGACCTAGAGTACTAAGCTCGAGCTTAGTACTCTAGGTCTGTCCTTTTTG-3′, eIF4G1 shRNA-1: 5′-CCGGCGGATGTTCTTTGATGCTCTACTCGAGTAGAGCATCAAAGAACATCCGTTTTTG-3′, eIF4G1 shRNA-2: 5′-CCGGGCGATGTGTCTTGAGCTAATACTCGAGTATTAGCTCAAGACACATCGCTTTTTG-3′, eIF4G1 shRNA-3: 5′-CCGGCACCGAGAACATATTAAAGTACTCGAGTACTTTAATATGTTCTCGGTGTTTTTG-3’.

### Measurement of bulk translation

For polysome profiling, translation was inhibited by the addition of 50 µg/mL cycloheximide for 5 min at 37 °C in culture media. The cells were then washed with PBS, trypsinized and resuspened in culture media. The cells were pelleted and washed with PBS prior to lysis in polysome lysis buffer (20 mM Tris pH 7.26, 130 mM KCl, 10 mM MgCl_2_, 2.5 mM DTT, 0.5% NP-40, 0.2 mg/mL heparin, 0.5% sodium deoxycholate, 50 µg/mL cycloheximide and 200 units/mL RNasin (Invitrogen)). Lysis occurred over 10 min on ice prior to clarification at 8000 *g* for 10 min at 4 °C. The absorbance at 260 nm was determined for each sample and an equal number of absorbance units for each sample was overlaid onto a 10–50% sucrose gradient made with sucrose gradient buffer (10 mM Tris pH 7.26, 60 mM KCl, 10 mM MgCl_2_, 1 mM DTT, 0.1 mg/mL heparin, 10 µg/mL cycloheximide). The gradients were subjected to ultracentrifugation at 36,000 rpm for 3 h at 4 °C. A Teledyne ISCO fractionation system with UV detector was used to determine absorbance at 254 nm along the gradient.

For measurement of translation rates by puromycin incorporation, MEFs were treated with 10 µM puromycin at room temperature for the times indicated. As a control, cells were also treated with 100 µg/mL cycloheximide for 5 min at 37 °C prior to addition of puromycin. Immediately following treatment, the cells were washed with 1 × PBS containing 100 µg/mL cycloheximide to inhibit further puromycin labeling. The cells were harvested and lysed in RIPA buffer with 1 × HALT (Pierce) and 100 µg/mL cycloheximide. Immunoblot was performed as described below with anti-puromycin antibody (Developmental Studies Hybridoma Bank, University of Iowa).

### Plasmid construction

The 5′-TOP reporter for mouse *Rpl23a* was made by PCR amplification of a region encompassing 500 bp upstream of the annotated transcription start site to the start codon using primers RPL23A Promoter Forward: 5′- GTACCTCGAGGAGCTATAAAGGGAAACCCTGTCTC -3′ and RPL23A 5′UTR Reverse: 5′- GTACCCATGGTGCTTGGCTGAAAAGGATGGCCC-3′. The PCR product was digested with XhoI and NcoI and ligated into pGL3-control (Promega). The resulting plasmid, pGL3-RPL23A-FF, was confirmed by Sanger sequencing.

To make pGL3-Renilla-deltaFF, Renilla luciferase was PCR amplified from pMT-DEST48-FLP^45^ with Renilla Luciferase Forward: 5′-TGGAAGCTTGGCATTCCGGTACTGTTGGTAAAGCCACCATGACTTCGAAAGTTTATG-3′ and Renilla Luciferase Reverse: 5′-TGGAAGCTTTTATTATTGTTCATTTTTGAGAAC-3′ and digested with HindIII (New England Biolabs, NEB). Renilla luciferase was then ligated into pGL3-Control digested HindIII (NEB) to make pGL3-Renilla-FF. To remove the firefly luciferase coding sequence, the plasmid was digested with NarI and XbaI (NEB), subsequently the ends were blunted with Klenow (NEB) and the plasmid was ligated. The resulting plasmid pGL3-Renilla-deltaFF was confirmed by Sanger sequencing.

### Transfection of MEFs and luciferase assay

The EEF2 (pIS1-Eef25UTR-renilla) and EEF2-mutant (pIS1-Eef25UTR-TOPmut-renilla) reporters were purchased from Addgene and have been described previously^26^. The day before transfection 1 × 10^5^ cells were plated per well in a six well dish. For transfection of the EEF2 reporters, the cells were transfected with Fugene6 (Promega) and 1 µg each of either pIS1-Eef25UTR-renilla or pIS1-Eef25UTR-TOPmut-renilla and pGL3-Control. After 24 h the cells were washed briefly with 1 × PBS prior to measurement of luciferase activity by Dual Luciferase Reporter Assay (Promega). For transfection of the RPL23A or SV40 reporters, the cells were transfected with Fugene6 and 1 µg each of either pGL3-control (firefly luciferase with SV40 promoter) or pGL3-RPL23A and pGL3-Renilla-deltaFF. After 24 h the cells were washed briefly with 1 × PBS prior to measurement of luciferase activity by Dual Luciferase Reporter Assay.

### Ribosome profiling and RNAseq

For RNAseq, total RNA was isolated from the appropriate cells using the Direct-zol RNA MiniPrep (Zymo) with Trizol (Invitrogen) for initial cell lysis. Contaminating genomic DNA was removed by treatment with Turbo DNA-free Kit per the manufacturers protocol (Invitrogen). The RNA concentration was determined by Qubit RNA BR Assay (Invitrogen) and ribosomal RNA was removed using the RiboZero Gold rRNA Removal Kit (Illumina) per the manufacturers protocol.

The ribo-depleted RNA (10 µL) was then fragmented by adding one volume of 2 × Fragmentation Buffer (0.5 M EDTA, 0.1 M Na_2_CO_3_, 0.1 M NaHCO_3_) and incubating at 95 °C for 20 min. Fragmentation was inhibited by the addition of 280 µL of Stop Solution (3 M NaOAc pH 5.5, 15 mg/mL GlycoBlue (Invitrogen)). The RNA was the precipitated by the addition of ethanol. The RNA was resuspended in 10 mM Tris pH 8.0 and resolved on a 15% acrylamide Urea-TBE gel (Bio-Rad). A region containing fragments between 17–34 nt (the same size as those isolated for ribosome profiling^46^) was excised and extracted overnight in RNA Extraction buffer (0.3 M NaOAc pH 5.5, 1 mM EDTA and 0.25% Sodium dodecylsulfate). The extracted RNA was precipitated by ethanol precipitation and subsequently used for sequencing library preparation as described below.

Ribosome footprinting was performed as described previously^46^, with the use of RNase T1 (Thermo Scientific) instead of RNase I for the footprinting step, see Supplemental Fig. 2a. The lysates were treated with 700 units of RNase T1 for 1 h at room temperature with gentle mixing. For both ribosome profiling and RNAseq, a small amount of yeast lysate was spiked-in to the lysis buffer for later normalization. We chose not to use the yeast spike-in for normalization as the replicate-to-replicate comparisons were weak (data not shown).

Following purification of the RNA footprints, sequencing library production was carried out for both the fragmented total RNA and ribosome footprints using the previously described protocol^46^. The RNAseq and ribosome profiling libraries were multiplexed and sequenced on a HiSeq 3000 (Illumina) by the Washington University Genome Technology Access Center.

### Data and code availability

Raw sequencing reads are available at https://www.ncbi.nlm.nih.gov/geo (GSE156749). Scripts used for analysis of differential expression and TE are available at GitHub (https://github.com/cottrellka/ARF_5TOP). The *DESeq2* results (see below) for Fold Change of TE and RNA are available in the Supplemental Dataset.

### Analysis of sequencing data

The sequences for the linkers and reverse transcriptase primer used in this study are available in the Supplementary Information. The ribosome profiling and RNAseq reads were processed to remove short reads, reads lacking the sequencing adapter and adapter only reads using *fastx_clipper*^47^ (fastx_clipper -a AGATCGGAAGAGCACACGTCTGAA -c -n -v -l 13). Reads were split by barcodes introduced by the linkers using fastx_barcode_spliter (example: –bcfile Barcodes.txt –prefix/Results/–suffix_ribo.fastq–eol < riboseq_trimmed_umi_trimmed.fastq). Barcodes and other sequences introduced during library production were removed by *umi-tools*^48^ (to remove UMIs: umi_tools extract –bc-pattern = NNNNNXXXXX –3prime) and *cutadapt* (to remove barcodes: cutadapt –cut -5)^49^. Sequencing reads mapping to rRNA or ncRNAs were removed using *Bowtie2*^50^ (example: bowtie2 rrna_seqs results.fastq –un results_norrna.fastq -S norrna_results). The processed reads were then aligned to the mouse genome (UCSC, mm10) using *TopHat*^51^ (example: tophat –no-novel-juncs –output-dir dir/ –GTF genes.gtf mm10/Sequence/Bowtie2Index/genome results_norrna_notrna_noncrna.fastq)*.* Read counts of those aligning to the coding sequence of each gene were then determined by *HTSeq*^52^ (example: htseq-count -t CDS accepted_hits.sam genes.gtf > htseq_results.txt). Differential TE was determined using *DESeq2* in which the design included an interaction term for assay and condition (~ assay + condition + assay:condition), this allows for determining differential TE via a ratio of ratios (treatment_RiboSeq/control_Riboseq)/(treatment_RNAseq/control_RNAseq)^32,53^ (see GitHub repository above for scripts used).

For classification of 5′-TOP genes, we used a previously described list of known 5′-TOP genes which is available in Supplementary Information^21^.

Gene ontology (GO) analysis was performed using the PANTHER Over Representation test via web browser http://geneontology.org/^54–56^. For this analysis, the annotation file used was GO version: 2020–03-23. Significance was determined by Fisher Exact test and multiple-testing correction was performed by FDR. Genes with fold change of TE > 0 and FDR adjusted p-value of < 0.05 where used to identify enriched GO terms.

### Immunoblot

Cell pellets were lysed and sonicated in RIPA Buffer (50 mM Tris pH 7.4, 150 mM NaCl, 1% Triton X-100, 0.1% sodium dodecyl sulfate and 0.5% sodium deoxycholate) with 1 × HALT Protease Inhibitor (Pierce). Seventy-five micrograms of protein lysate were resolved on 4–12% TGX Acrylamide Stain-Free gels (Bio-Rad). Stain-Free gels were imaged prior to transfer to PVDF membrane (Millipore). The blots were then probed with the appropriate primary antibodies: Abcam PABP (ab21060); Bethyl—GAPDH (A300-641A), LARP1 (A302-087A), EEF2 (A301-688A), RPL23A (A303-932A-T); Cell Signaling Technologies—TPT1(5128), p-MTOR (2971), p-S6 (2215S), p-S6K (9205S), p-4EBP1 (2855S), eIF4G1 (2858), p53 (2524S), eIF4E (9742S), 4EBP1 (9452S); Santa Cruz Biotechnology—ARF (sc-32748), RPL22 (sc-136413), University of Iowa, DSHB—puromycin. Primary antibodies were detected with horseradish-peroxidase conjugated secondary antibodies (Jackson ImmunoResearch) and detection was carried out with Clarity Western ECL Substrate (Bio-Rad).

### Quantitative PCR

Total RNA was isolated using the Nucleospin RNA (Macherey–Nagel) with on column DNase treatment. Reverse transcription to make cDNA was performed with iScript Supermix (Bio-Rad). For qPCR the primers listed in the Supplementary Information were used with iTaq SYBR Green (Bio-Rad). Fold change in RNA expression was determined by the ΔΔCt method with normalization to PSMA5, TOMM20 and ATP5B. The normalization genes were chosen based on steady expression across all samples as measured by RNA-seq. For qPCR of the luciferase reporters: RNA was treated with the restriction enzyme AluI for 30 min at 37 °C in 1 × Turbo DNase Buffer (Thermo Fisher). After treatment 1 µL of Turbo DNase (Thermo Fisher) was added and remaining DNA was digested for 30 min at 37 °C. Turbo DNase was removed using the Turbo DNA-Free Kit (Thermo Fisher) and the RNA was used for cDNA synthesis as above, with the addition of a 20 min incubation at 80 °C following first strand synthesis to inhibit the restriction enzyme. This approach allowed for complete removal of plasmid DNA from the RNA sample as determine by qPCR of a no-reverse transcriptase control reaction (data not shown). Quantitative PCR was performed as above using *Renilla* and firefly luciferase qPCR primers (Supplementary Information). Fold change in firefly (RPL23A reporters) or *Renilla* (EEF2 reporters) luciferase RNA was determined by the ΔΔCt method with normalization to *Renilla* (RPL23A reporters) or firefly (EEF2 reporters) luciferase, respectively.

## Supplementary Information

Supplementary Information 1.

Supplementary Information 2.

Supplementary Information 3.

Supplementary Information 4.

Supplementary Information 5.
