# Supplementary material for: Upregulation of 5′-terminal oligopyrimidine mRNA translation upon loss of the ARF tumor suppressor
Source: Sci Rep. 2020 Dec 17;10:22276. doi: 10.1038/s41598-020-79379-8 (PMC7747592; doi:10.1038/s41598-020-79379-8)
Supplement: Supplementary file 1 — Supplementary Information 1. [file 41598_2020_79379_MOESM1_ESM.docx]

**Supplementary Information**

**Upregulation of 5’-terminal oligopyrimidine mRNA translation upon loss of the ARF tumor suppressor**

Kyle A. Cottrell^1^, Ryan C. Chiou^1^, Jason D. Weber^1,2,*^

^1^Department of Medicine, Division of Molecular Oncology and ^2^Department of Cell Biology and Physiology, Siteman Cancer Center, Washington University School of Medicine, Saint Louis, Missouri, USA

*Corresponding author

Correspondence:

Jason D. Weber, Ph.D.

Department of Medicine

Division of Molecular Oncology

Washington University School of Medicine

660 South Euclid Avenue

Campus Box 8069

St. Louis, MO 63110 USA

Email: [jweber@wustl.edu](mailto:jweber@wustl.edu)

Telephone: 314-747-3896

Fax: 314-362-0152

**Description of Supplementary Datasets:**

**S1 - DESeq2 Knockout TE –** Results of DESeq2 analysis for differential translation efficiency (TE) comparing WT-shSCR MEFs to *Arf^-/-^* **-**shSCR MEFs.

**S2 - DESeq2 Knockdown TE –** Results of DESeq2 analysis for differential translation efficiency (TE) comparing WT-shSCR MEFs to WT-shARF MEFs.

**S3 - DESeq2 Knockout RNA –** Results of DESeq2 analysis for differential RNA expression comparing WT-shSCR MEFs to *Arf^-/-^* **-**shSCR MEFs.

**S4 - DESeq2 Knockdown RNA –** Results of DESeq2 analysis for differential RNA expression comparing WT-shSCR MEFs to WT-shARF MEFs.

**Quantitative PCR Primers (5’ -> 3’)**

PSMA5 qF

ATTGGCTCTGCTTCTGAGGG

PSMA5 qR

ATGAGCGAGGACTTGATGGC

TOMM20 qF

TTACAGCAGACTCTTCCGCC

TOMM20 qR

TCTTCAGCCAAGCTCTGAGC

ATP5B qF

TGCAGGAAAGGATCACCACC

ATP5B qR

AATAGCCCGGGACAACACAG

Renilla-qF

ATAACTGGTCCGCAGTGGTG

Renilla-qR

TAAGAAGAGGCCGCGTTACC

Firefly-qF

TCCATCTTGCTCCAACACCC

Firefly-qR

TCATCGTCTTTCCGTGCTCC

LARP1 qF

TCCTTTCACCGTGTACAGGC

LARP1 qR

TGCGGACTTTCTCCTCAACC

RPL22 qF

TGAAGTTCACCCTGGACTGC

RPL22 qR

GGTTGCCAGCTTTCCCATTC

PABPC1 qF

GTTACCATGCAACAGCCTGC

PABPC1 qR

GAAACAGCCGTTCACCCAAC

TPT1 qF

AGCCATGACGAGCTGTTCTC

TPT1 qR

TTTCCACCGATGAGCGAGTC

RPL23A qF

AAGATCCGAACGTCACCCAC

RPL23A qR

CAAGCTTGTTTCTCCTGGGC

EEF2 qF

AGCGGGCCAAGAAAGTAGAG

EEF2 qR

GCTGACTTGCTGAACTTGCC

**Ribosome profiling and RNaseq Oligos**

(linkers: bold indicates sample barcode used for multiplexing, underlined is UMI which was removed from sequencing reads prior to mapping)

**Linker 1**

5^′^-/5Phos/NNNNN**ATCGT**AGATCGGAAGAGCACACGTCTGAA/3ddC/

**Linker 2**

5^′^-/5Phos/NNNNN**AGCTA**AGATCGGAAGAGCACACGTCTGAA/3ddC/

**Linker 3**

5^′^-/5Phos/NNNNN**CGTAA**AGATCGGAAGAGCACACGTCTGAA/3ddC/

**Linker 4**

5^′^-/5Phos/NNNNN**CTAGA**AGATCGGAAGAGCACACGTCTGAA/3ddC/

**Linker 5**

5^′^-/5Phos/NNNNN**GATCA**AGATCGGAAGAGCACACGTCTGAA/3ddC/

**Linker 6**

5^′^-/5Phos/NNNNN**GCATA**AGATCGGAAGAGCACACGTCTGAA/3ddC/

**Reverse transcriptase primer**

(the beginning bases ‘RN’ were removed from sequencing reads prior to mapping)

5^′^-/5Phos/RNAGATCGGAAGAGCGTCGTGTAGGGAAAGAG/iSp18/GTGACTGGAGTTCAGACGTGTGCTC

**Library construction primers:**

**Illumina Adapter F**

5’-AATGATACGGCGACCACCGAGATCTACACTCTTTCCCTACACGACGCTC-3’

**Illumina Adapter R 1 (used for Ribosome profiling)**

5’-CAAGCAGAAGACGGCATACGAGATCGTGATGTGACTGGAGTTCAGACGTGTG-3’

**Illumina Adapter R 2 (used for RNAseq)**

5’-CAAGCAGAAGACGGCATACGAGATACATCGGTGACTGGAGTTCAGACGTGTG-3’

**Known 5’-TOP genes**

Eef1a1

Eef1b2

Eef2

Hnrnpa1

Npm1

Pabpc1

Rpl10

Rpl10a

Rpl11

Rpl12

Rpl13

Rpl13a

Rpl14

Rpl15

Rpl17

Rpl18

Rpl18a

Rpl19

Rpl21

Rpl22

Rpl23

Rpl23a

Rpl24

Rpl26

Rpl27

Rpl27a

Rpl28

Rpl29

Rpl3

Rpl30

Rpl31

Rpl32

Rpl34

Rpl35

Rpl35a

Rpl36

Rpl36a

Rpl37

Rpl37a

Rpl38

Rpl39

Rpl4

Rpl5

Rpl6

Rpl7

Rpl8

Rpl9

Rplp0

Rplp1

Rpp2

Rps10

Rps11

Rps12

Rps13

Rps14

Rps15

Rps15a

Rps16

Rps18

Rps19

Rps2

Rps20

Rps21

Rps23

Rps24a

Rps25

Rps26

Rps27

Rps28

Rps29

Rps3

Rps3a

Rps4x

Rps5

Rps6

Rps7

Rps8

Rps9

Rpsa

Tpt1

Rpl41

Rpl7a


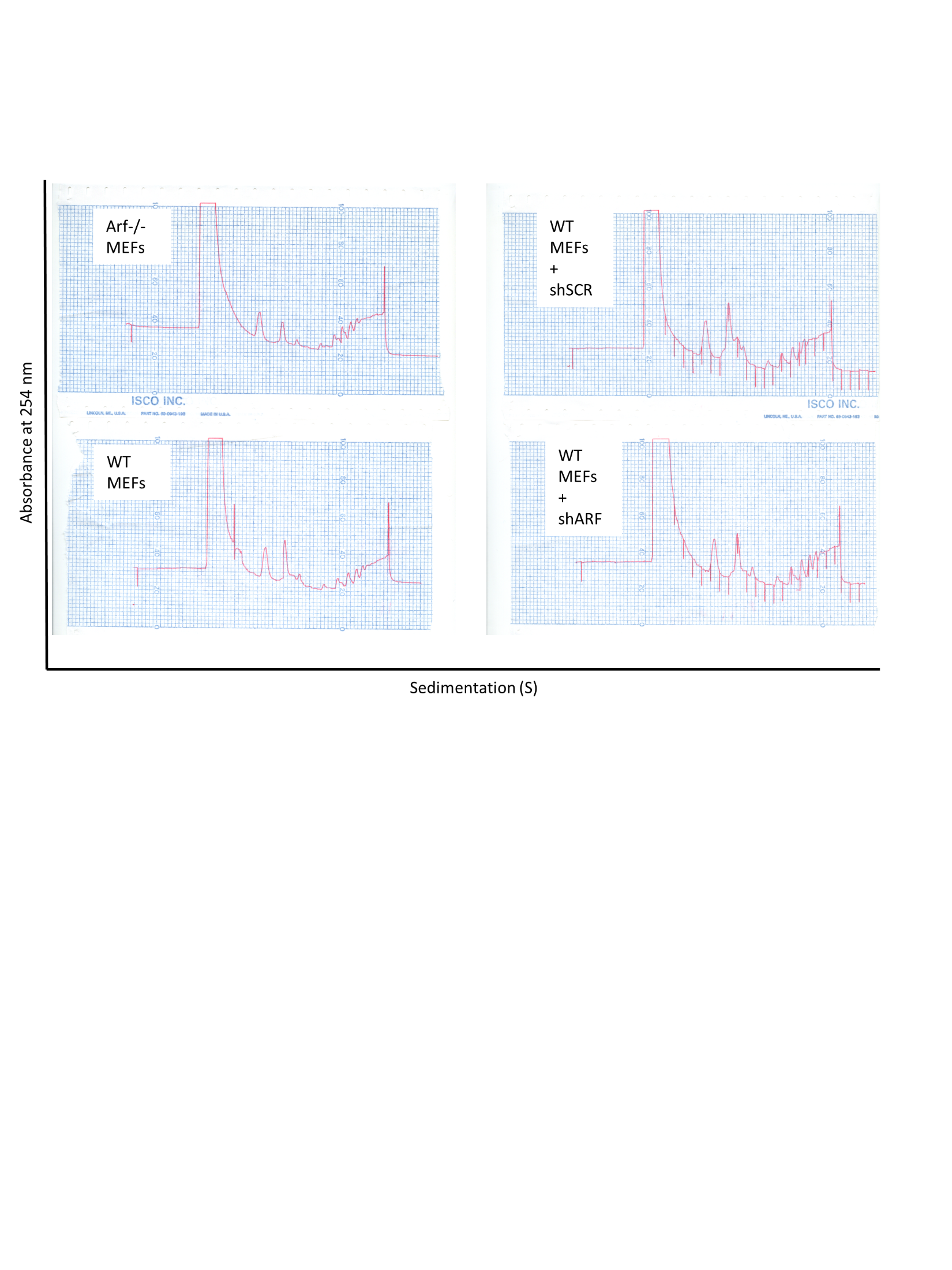


**Supplementary Figure 1: Raw polysome profiling traces for ARF knockout and knockdown MEFs** The analog polysome profiling traces on the left were digitally traced in Microsoft PowerPoint to make panel **a** in Figure 1 of the main text. The sudden spike in the WT MEFs trace corresponds to a bubble passing through the detector.


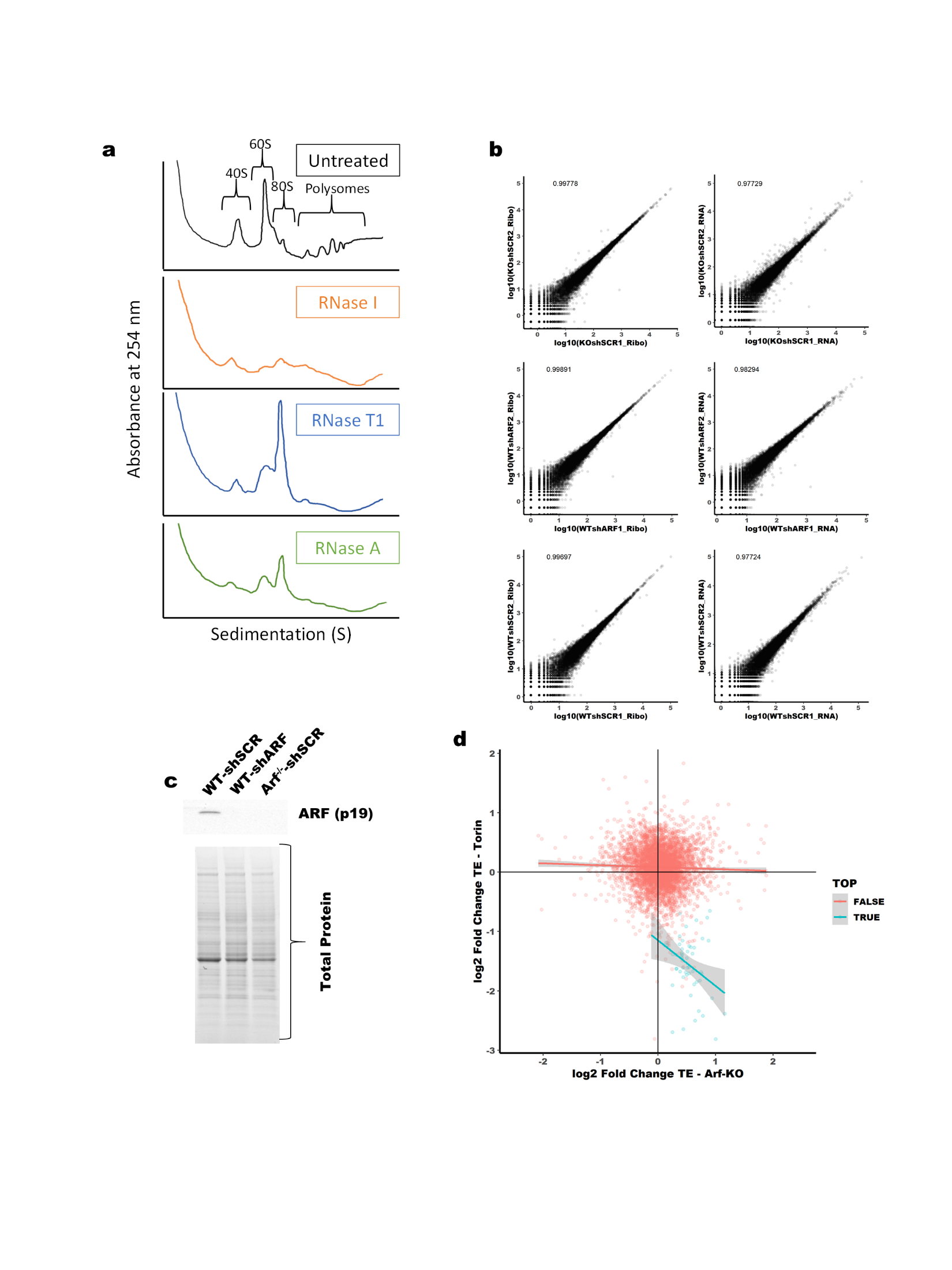


r = -0.35

p-value = 0.01112

**Supplementary Figure 2: Ribosome profiling nuclease selection and reproducibility** Panel **a** shows the effect of ribonuclease treatment of MEF lysates using RNase I, T1 or A. RNase T1 completely removed polysome peaks while maintaining a strong monosome peak. **b** Replicate-to-replicate comparisons of ribosome profiling and RNAseq normalized read counts per gene. **c** uncropped image from Figure 2a. **d** Scatter plot showing comparison between fold change of TE following knockout of *Arf*  or treatment of *p53^-/-^* MEFs with Torin (data from ^1^). The blue points are known 5’-TOP genes.


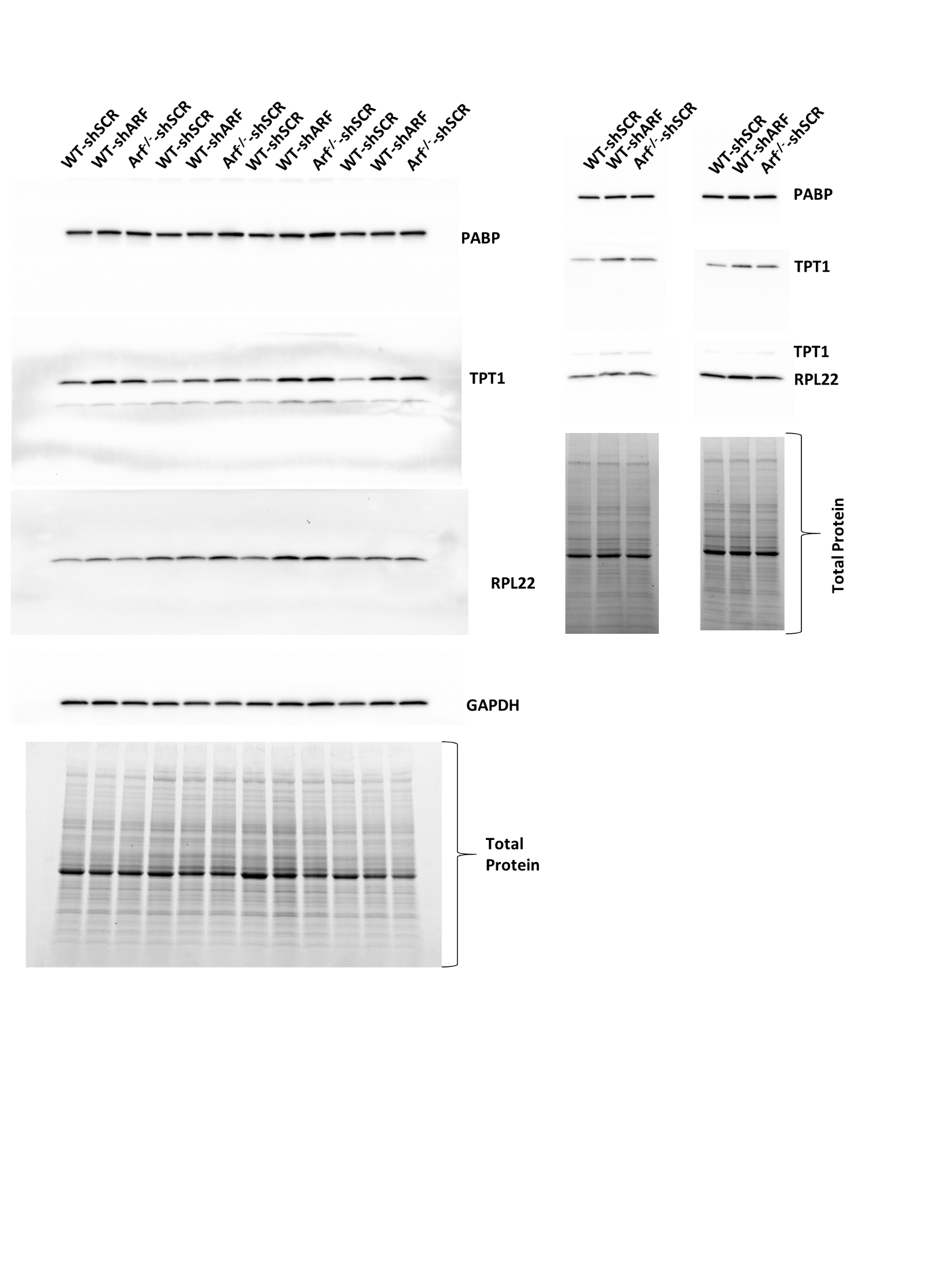


**Supplementary Figure 3: Extended data for Figure 3** Uncropped immunoblots for Figure 3. For the right two panels, the TPT1 blot was re-probed with an antibody for RPL22.


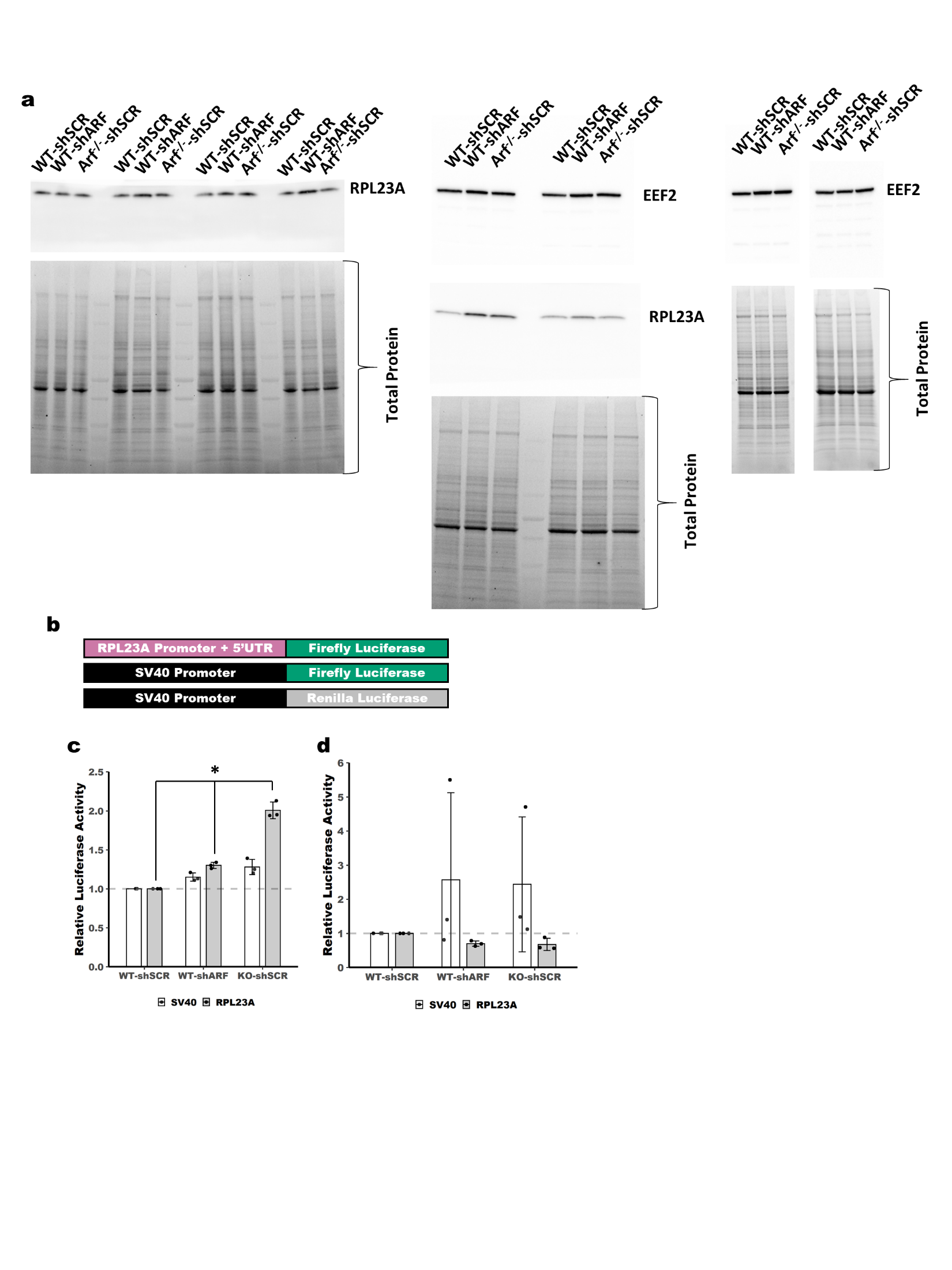


**Supplementary Figure 4: Extended data for Figure 3** Panel **a** Uncropped immunoblots for Figure 3. **b** Schematic of the luciferase reporters used in panels **c** and **d. c** Luciferase activity of a RPL23A reporter is increased in ARF-null MEFs. Luciferase activity was normalized to *Renilla* luciferase transfection control and set relative to WT-shSCR. Mean±SD, n=3. d qPCR shows no increase in mRNA expression of the RPL23a reporter. Normalized to *Renilla* luciferase transfection control. Mean±SD, n=3. * *p*-value < 0.05; two-tailed t-test with Bonferoni correction


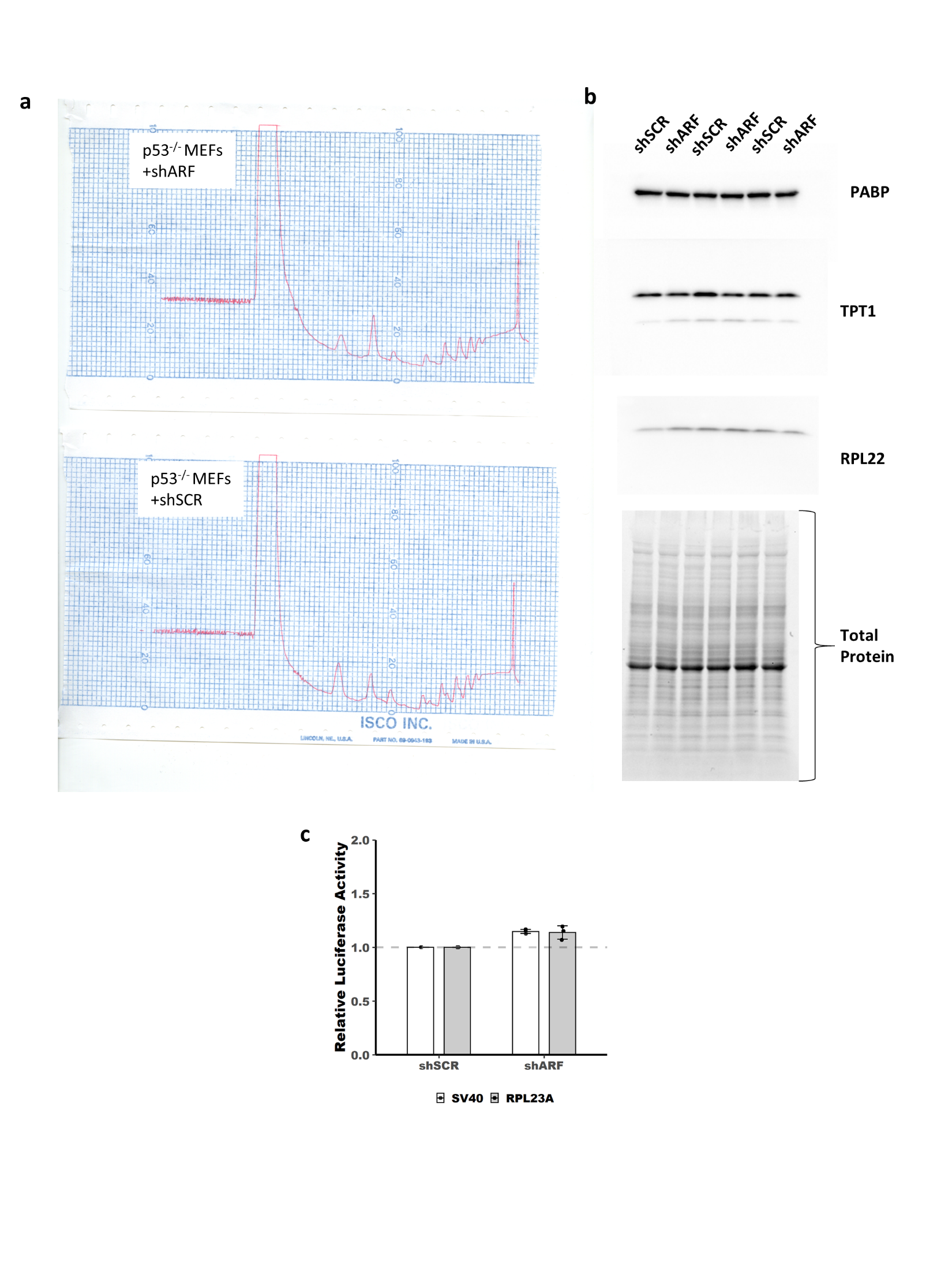


**Supplementary Figure 5: Source images for Figure 4** Panel **a** Analog polysome profiling trace used in Figure 4. **b** uncropped immunoblots for Figure 4. See main text for further details. **c** Knockdown of ARF in *p53^-/-^* MEFs has no effect of luciferase reporter activity for a reporter containing either the RPL23A 5’UTR or a control 5’UTR (vector sequence of pGL3-Control) driven by the SV40 promoter.


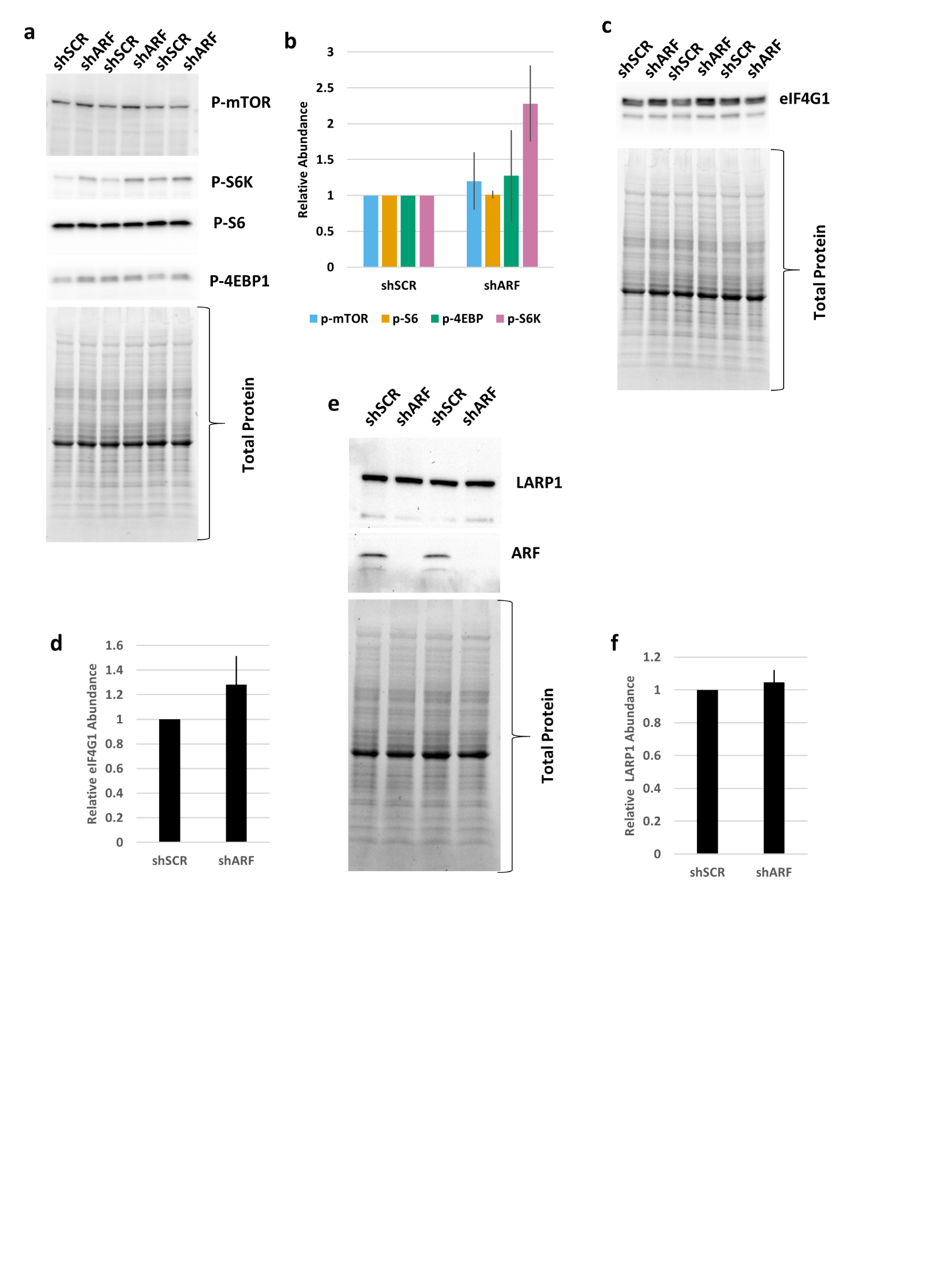


**Supplementary Figure 6: Analysis of 5’-TOP regulators expression and activity following knockdown of ARF in *p53^-/-^* MEFs** Panel **a** assessment of mTORC1 activity following knockdown of ARF in *p53^-/-^* MEFs. **b** Quantitation of the immunoblot shown in panel **a**. Mean +/- standard deviation, N = 3. **c** assessment of eIF4G1 expression following knockdown of ARF in *p53^-/-^* MEFs. **d** Quantitation of the immunoblot shown in panel **c**. Mean +/- standard deviation, N = 3. **e** assessment of LARP1 expression following knockdown of ARF in *p53^-/-^* MEFs. **f** Quantitation of the immunoblot shown in panel **e**, Mean +/- standard deviation, N = 2.


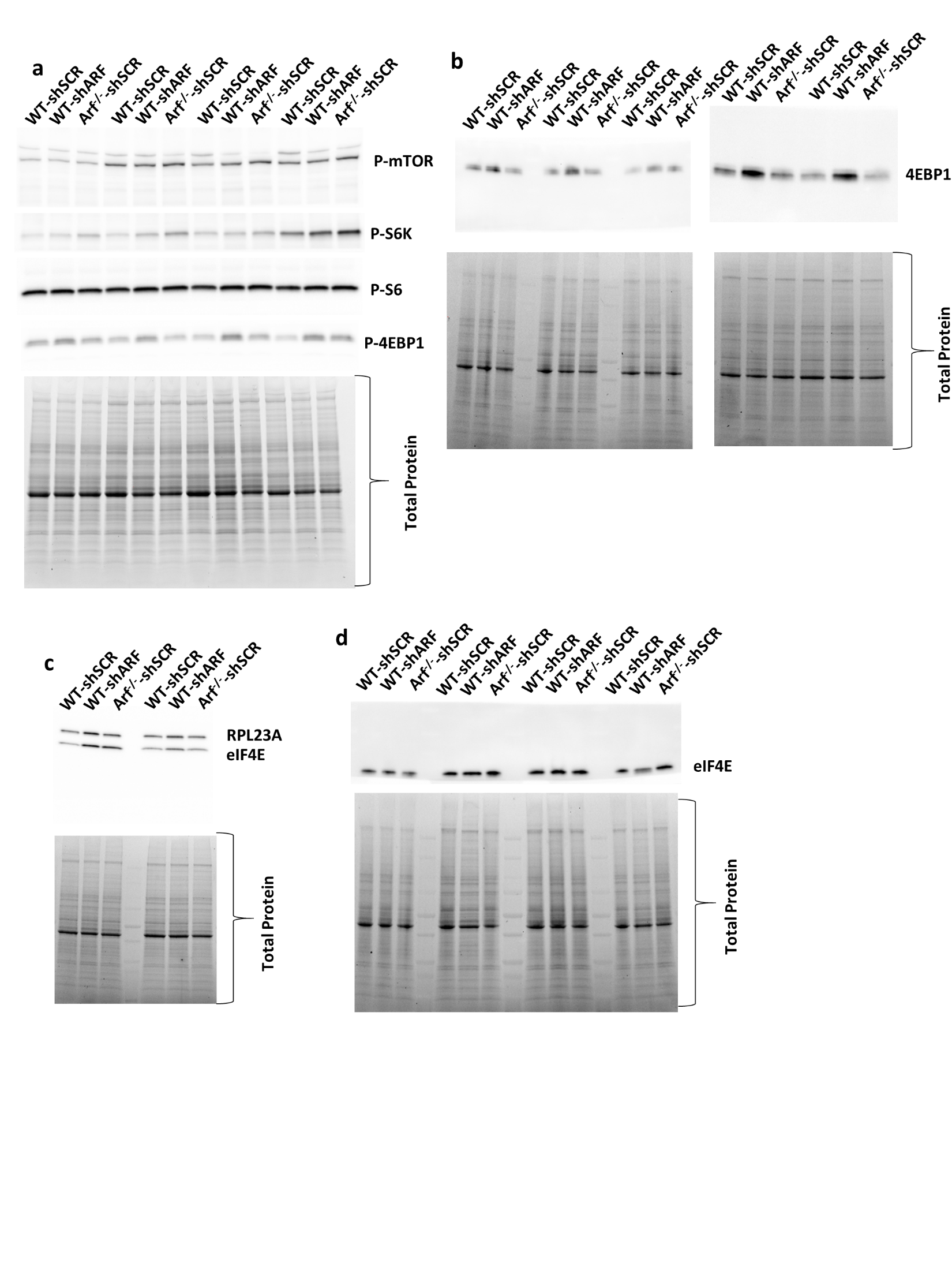


**Supplementary Figure 7: Source Images for Figure 5** Uncropped immunoblots from **Figure 5**. See main text for further details. **a-d** source for Figure 5a. For panel **c**, the blot from the middle panel of Supplementary Figure 5a was re-probed with an antibody for eIF4E, as such, the total protein gel image is the same.


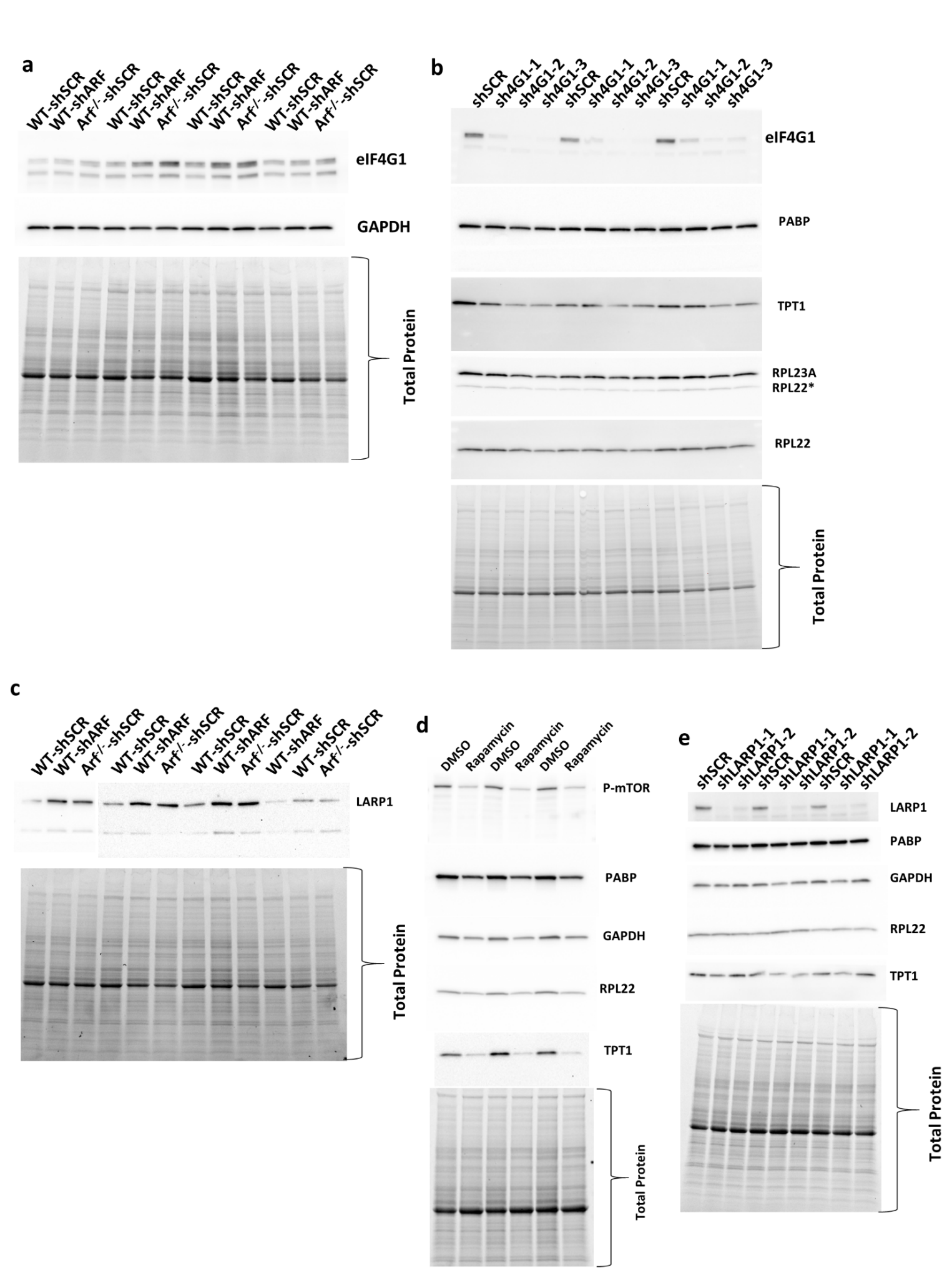


**Supplementary Figure 8: Source Images for Figure 5** Panel **a** source for Figure 5e, **b** source for Figure 5g, **c** source for Figure 5i, **d** source for Figure 5c and **e** source for Figure 5l**.** For panel c, replicate 1 for LARP1 was repeated due to a smear upon transfer and was ran a second time, this is the cause of the composite panel.


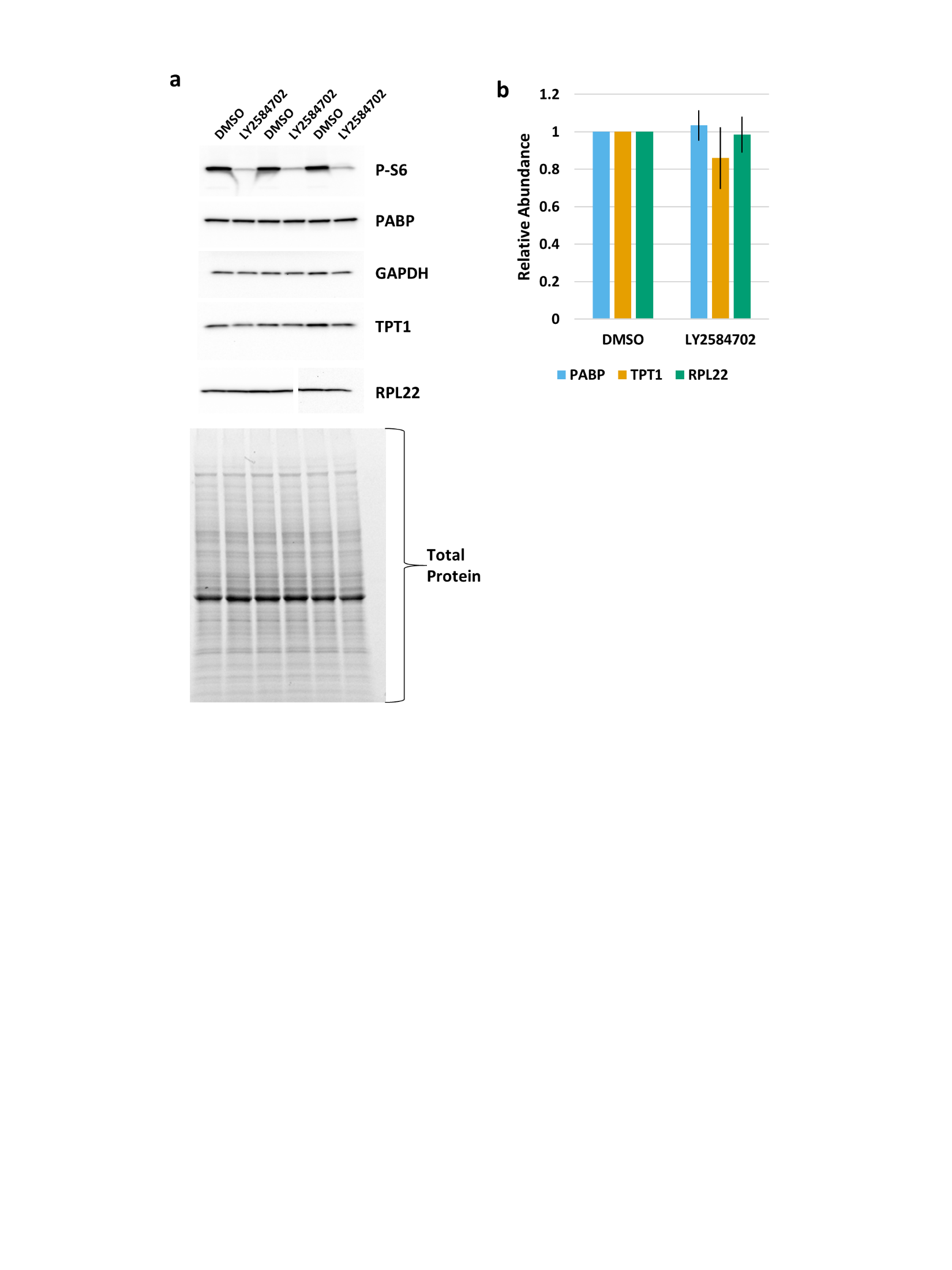


**Supplementary Figure 9: S6-Kinase inhibition has no effect on 5’-TOP mRNA expression and overexpression of eIF4G1** *Arf^-/-^* MEFs were treated with LY2584702 (ApexBio Technology) at a concentration of 1 µM for 4 days prior to harvesting. **a** Immunoblot analysis of 5’-TOP mRNA encoded proteins and P-S6 following treatment, quantified in panel **b**. Mean +/- standard deviation, n = 3. For RPL22 – the replicate 3 sample smeared upon transfer and was ran a second time, this is the cause of the composite panel.


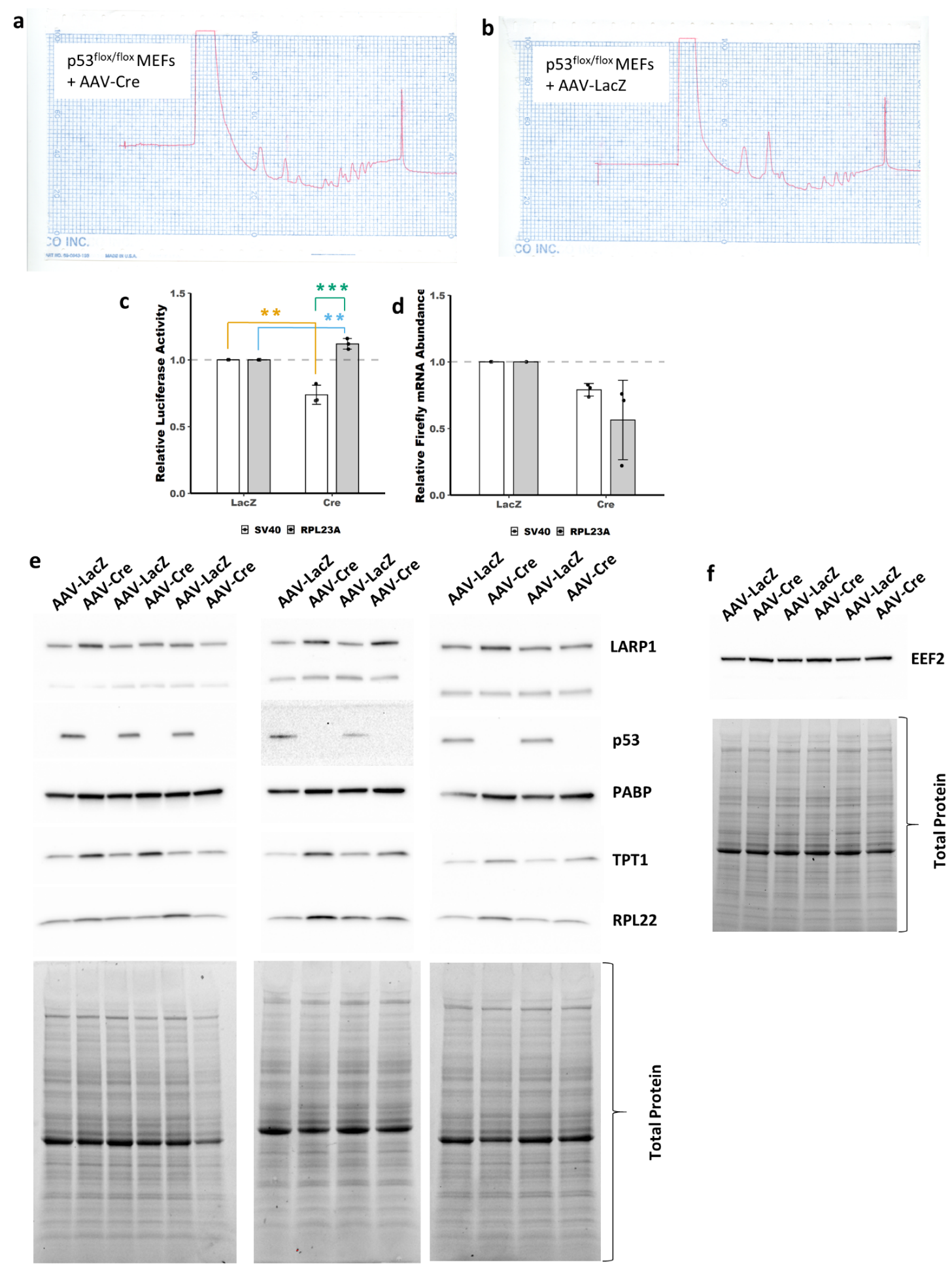
**Supplementary Figure 10: Source images for Figure 6** Panels **a** and **b**, analog trace used in Figure 6a. **c** Luciferase activity of a 5’-TOP reporter is increased in *p53^-/-^* MEFs. Luciferase activity was normalized to *Renilla* luciferase transfection control and set relative to LacZ control. Mean±SD, n=3. **d** qPCR shows no increase in mRNA expression of the RPL23a reporter. Normalized to *Renilla* luciferase transfection control. Mean±SD, n=3. * *p*-value < 0.05; two-tailed t-test with Bonferoni correction **e** and **f** uncropped immunoblots for Figure 6b. See main text for further details.


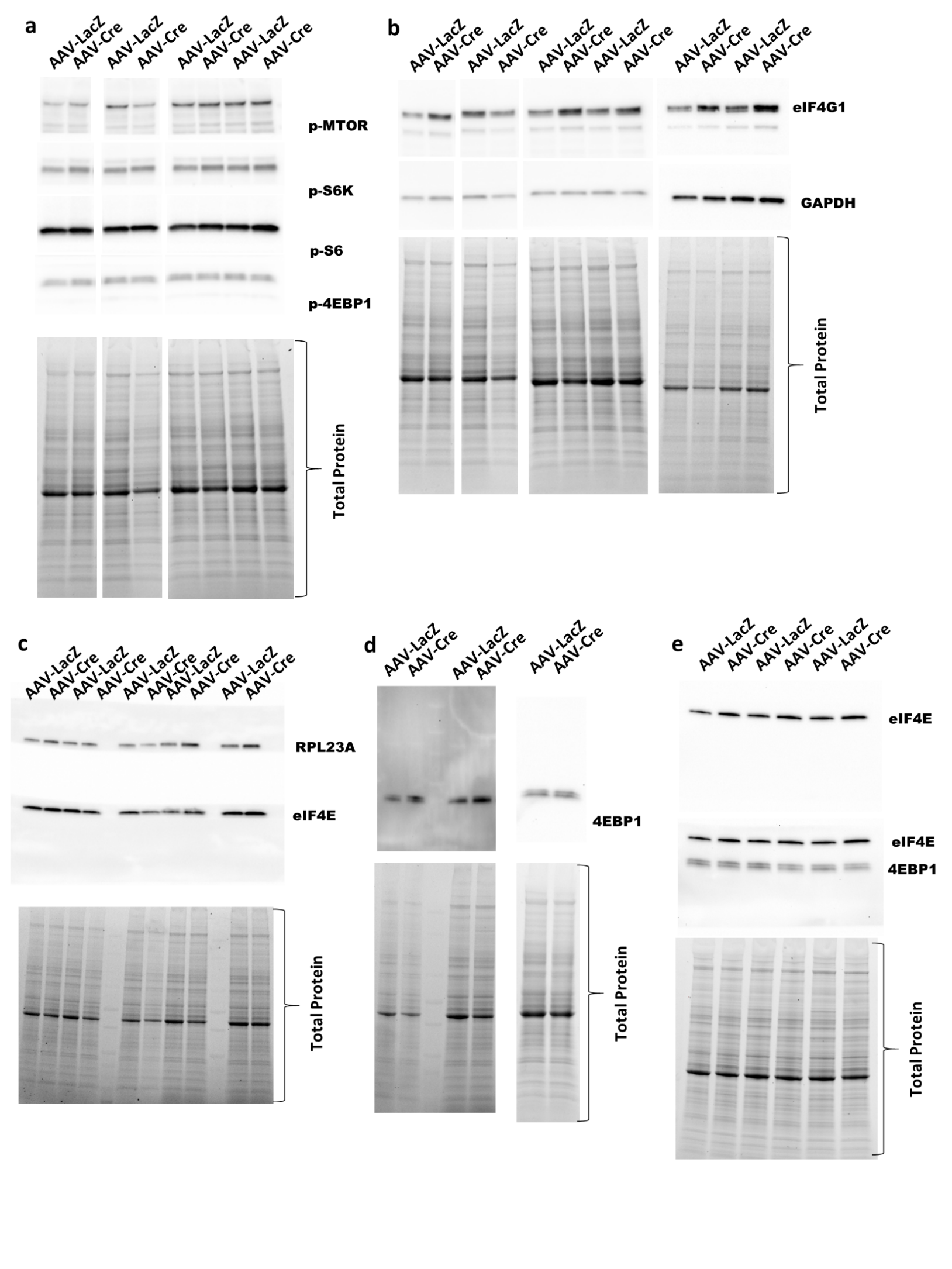


**Supplementary Figure 11: Source images for Figure 6** Panels **a** – **e** are uncropped immunoblots for Figure 6b. For panel **e** the upper blot (eIF4E) was re-probed with an antibody for 4EBP1.

1 Thoreen, C. C. *et al.* A unifying model for mTORC1-mediated regulation of mRNA translation. *Nature* **485**, 109-113, doi:10.1038/nature11083 (2012).
